# Spatiotemporal and Seasonal Trends of Class A and B Notifiable Infectious Diseases in China: Retrospective Analysis

**DOI:** 10.2196/42820

**Published:** 2023-04-27

**Authors:** Junyao Zheng, Ning Zhang, Guoquan Shen, Fengchao Liang, Yang Zhao, Xiaochen He, Ying Wang, Rongxin He, Wenna Chen, Hao Xue, Yue Shen, Yang Fu, Wei-Hong Zhang, Lei Zhang, Samir Bhatt, Ying Mao, Bin Zhu

**Affiliations:** 1 China Institute for Urban Governance Shanghai Jiao Tong University Shanghai China; 2 School of International and Public Affairs Shanghai Jiao Tong University Shanghai China; 3 School of Public Policy and Administration Xi'an Jiaotong University Xi'an China; 4 School of Public Administration and Policy Renmin University of China Beijing China; 5 School of Public Health and Emergency Management Southern University of Science and Technology Shenzhen China; 6 The George Institute for Global Health Peking University Health Science Center Beijing China; 7 WHO Collaborating Centre on Implementation Research for Prevention and Control of Noncommunicable Diseases Melbourne Australia; 8 Vanke School of Public Health Tsinghua University Beijing China; 9 Center for Chinese Public Administration Research and School of Government Sun Yat-sen University Guangzhou China; 10 Stanford Center on China's Economy and Institutions Stanford University Stanford, CA United States; 11 Laboratory for Urban Future School of Urban Planning and Design Peking University Shenzhen Graduate School Shenzhen China; 12 Department of public administration School of Government Shenzhen University Shenzhen China; 13 International Centre for Reproductive Health Department of Public Health and Primary Care Ghent University Ghent Belgium; 14 China-Australia Joint Research Center for Infectious Diseases School of Public Health Xi’an Jiaotong University Health Science Center Xi’an China; 15 Artificial Intelligence and Modelling in Epidemiology Program Melbourne Sexual Health Centre Alfred Health Melbourne Australia; 16 Central Clinical School Faculty of Medicine Monash University Melbourne Australia; 17 Department of Epidemiology and Biostatistics College of Public Health Zhengzhou University Zhengzhou China; 18 MRC Centre for Global Infectious Disease Analysis and the Abdul Latif Jameel Institute for Disease and Emergency Analytics School of Public Health, Imperial College London United Kingdom; 19 Section of Epidemiology Department of Public Health University of Copenhagen Copenhagen Denmark

**Keywords:** notifiable infectious diseases, spatial epidemiology, temporal trends, seasonal feature, spatial disparities

## Abstract

**Background:**

China is the most populous country globally and has made significant achievements in the control of infectious diseases over the last decades. The 2003 SARS epidemic triggered the initiation of the China Information System for Disease Control and Prevention (CISDCP). Since then, numerous studies have investigated the epidemiological features and trends of individual infectious diseases in China; however, few considered the changing spatiotemporal trends and seasonality of these infectious diseases over time.

**Objective:**

This study aims to systematically review the spatiotemporal trends and seasonal characteristics of class A and class B notifiable infectious diseases in China during 2005-2020.

**Methods:**

We extracted the incidence and mortality data of 8 types (27 diseases) of notifiable infectious diseases from the CISDCP. We used the Mann-Kendall and Sen’s methods to investigate the diseases’ temporal trends, Moran I statistic for their geographical distribution, and circular distribution analysis for their seasonality.

**Results:**

Between January 2005 and December 2020, 51,028,733 incident cases and 261,851 attributable deaths were recorded. Pertussis (*P*=.03), dengue fever (*P*=.01), brucellosis (*P*=.001), scarlet fever (*P*=.02), AIDS (*P*<.001), syphilis (*P*<.001), hepatitis C (*P*<.001) and hepatitis E (*P*=.04) exhibited significant upward trends. Furthermore, measles (*P*<.001), bacillary and amebic dysentery (*P*<.001), malaria (*P*=.04), dengue fever (*P*=.006), brucellosis (*P*=.03), and tuberculosis (*P*=.003) exhibited significant seasonal patterns. We observed marked disease burden–related geographic disparities and heterogeneities. Notably, high-risk areas for various infectious diseases have remained relatively unchanged since 2005. In particular, hemorrhagic fever and brucellosis were largely concentrated in Northeast China; neonatal tetanus, typhoid and paratyphoid, Japanese encephalitis, leptospirosis, and AIDS in Southwest China; BAD in North China; schistosomiasis in Central China; anthrax, tuberculosis, and hepatitis A in Northwest China; rabies in South China; and gonorrhea in East China. However, the geographical distribution of syphilis, scarlet fever, and hepatitis E drifted from coastal to inland provinces during 2005-2020.

**Conclusions:**

The overall infectious disease burden in China is declining; however, hepatitis C and E, bacterial infections, and sexually transmitted infections continue to multiply, many of which have spread from coastal to inland provinces

## Introduction

### Evidence Before This Study

China, the world’s most populous nation, has greatly reduced its infectious disease burden over the past 2 decades. There is tremendous interest in understanding the spatiotemporal trends of notifiable infectious diseases. We searched PubMed and the China National Knowledge Internet Database for all journal articles before August 1, 2022, using the following keywords: (“notifiable infectious diseases” OR “notifiable diseases” OR “notifiable infections”) AND (“China” OR “Chinese”) AND (“epidemiology” OR “epidemiological characteristics” OR “epidemiological features” OR “surveillance” OR “incidence” OR “morbidity” OR “mortality” OR “trends” OR “seasonality” OR “review”). Several studies have reviewed the national incidence and mortality data of notifiable infectious diseases in China. However, to date, previous studies have only assessed the temporal trends, and few studies have systematically reviewed and mapped out the disparities in infectious disease burden; further, the latest epidemiological characteristics after 2016 have not been explored.

### Added Value of This Study

To our knowledge, this study is the first to systematically explore and compare the spatiotemporal trends of different types of notifiable infectious disease cases and has covered the longest period since the establishment of the China Information System for Disease Control and Prevention (CISDCP). This study also identified the latest temporal trends and seasonal characteristics of different types of notifiable infectious diseases. Regional differences and identified high-risk areas can be observed on the descriptive and spatial cluster maps, indicating that some provinces are struggling to combat certain types of infectious diseases. Therefore, our results supplement those of previous studies and provide insights into evidence-based infectious disease prevention and control policy making in China.

### Implications of All the Available Evidence

There is no one-size-fits-all strategy for preventing and controlling infectious diseases in different administrative units (provinces, autonomous regions, and municipalities) due to huge disparities in the burdens of all the types of notifiable infectious diseases in China. Continuous efforts should be made through subtype-specific and region-oriented infectious disease prevention and control policies by prioritizing the most recently identified high-risk areas and high-risk seasons. A more detailed prevention and control plan should be developed based on the current trends and patterns of notifiable infectious diseases, which should aid in setting a clear goal for the management of each type of infectious disease, be renewable with time, target high-risk areas, and specify the duties of each sector.

### Background

Infectious disease prevention and control are key focuses of the Millennium Development Goals and Sustainable Development Goals [1] of the World Health Organization (WHO). Despite achievements in reducing infectious disease burden globally, including polio elimination and smallpox eradication, several recent major epidemics have significantly interrupted people’s lives and the country’s economic activities [2]. These include the 2003 SARS epidemic, 2009 influenza A virus epidemic, and 2013-2014 H7N9 Avian influenza epidemic, which have raised concerns regarding infectious diseases worldwide [3,4]. In recent years, the COVID-19 pandemic has resulted in over 756 million cases and over 6.8 million deaths (as of February 2023, data from the WHO), causing considerable long-term concern globally [5-7].

China has made considerable progress in the battle against infectious diseases and has witnessed a significant drop in the burden of many major infectious diseases [8]. However, infectious diseases remain a major health concern in China, where they still impose heavy burdens and economic losses nationwide [9] (major outbreaks of infectious diseases after SARS are listed in Table S1 in Multimedia Appendix 1). To respond to and control infectious disease outbreaks more effectively, the Chinese government has published a list of notifiable infectious diseases, allowing for adequate monitoring and early warning in case of possible outbreaks [10]. The *Law of the People’s Republic of China on the Prevention and Treatment of Infectious Diseases* [11] has mandated 40 infectious diseases (different types of viral hepatitis are regarded as 1 single disease under this law) with high incidences or severe consequences as legally notifiable to facilitate the tracking of their incidence and spread [12]. The notifiable infectious diseases in China are classified as A, B, or C based on their potential threat, with severity decreasing from A to C (group A includes 2 diseases, group B includes 27 diseases, and group C includes 11 diseases; Table S2 in Multimedia Appendix 1) [13]. Soon after the SARS epidemic, the Chinese government decided to build an internet-based CISDCP, which links hospitals, the Centers for Disease Control and Prevention (CDC), and health facilities at national, provincial, prefectural/city, and county/district levels and covers community health centers, township health centers, and village clinics [14]. The real-time reporting of notifiable infectious diseases through the CISDCP has reduced the underreporting of infectious diseases, thus improving infectious disease–related data reliability and accuracy following its implementation in 2003 [14].

Previous studies reviewing the epidemiology of notifiable infectious diseases in China tended to focus on epidemiological features and national trends in incidence. For instance, Zhang and Wilson [15] reviewed the national trends in notifiable infectious diseases in China from 1975 to 2008 and discussed the implications for public health interventions. Furthermore, 10 years after the SARS outbreak, Yang et al [12] reviewed the incidence trends of notifiable infectious diseases from 2005 to 2013, thereby providing evidence supporting an evaluation of China’s prevention and control policies. In addition, based on 132,858,005 included cases, Jiang et al [9] explored the 31-year epidemiological trends in notifiable infectious diseases from 1986 to 2016. In the aforementioned studies, despite the knowledge gained, little attention was paid to the geographical distribution patterns of infectious disease cases, limiting the understanding of infectious disease epidemiology. Although several studies have examined the geographical distribution of a single infectious disease or single type of disease [16], few have systematically compared the spatial disparities in the burdens of different subtypes of infectious diseases in China.

The lack of knowledge regarding how infectious disease incidence varies across geographical regions and disease types significantly hinders evidence-based policy design. To fill this knowledge gap, this study aimed to review the 16-year spatiotemporal and seasonal trends of 8 major types of class A and B notifiable infectious diseases (quarantinable diseases, vaccine-preventable diseases, gastrointestinal diseases, vector-borne diseases, zoonotic infections, bacterial infections, sexually transmitted infections, and viral hepatitis) after the establishment of the CISDCP in China. The study will add further value to the existing literature and provide additional evidence to inform stakeholders in formulating infectious disease prevention and control policies.

## Methods

### Data and Measurements

Annual incidence and mortality data recorded from 2005 to 2020 were extracted from the National Health Statistical Yearbooks [17] published by the National Health Commission of China. Incidence data in 31 provincial units (provinces, autonomous regions, and municipalities), which were exported from the CISDCP, were obtained from the Public Health Science Data Center of the Chinese CDC. In addition, we collected the monthly incidence (the number of incident cases per month divided by the population size) data of each disease between January 2005 and December 2020 from the CISDCP. Subsequently, the average monthly incidence of each infectious disease was calculated as the number of newly infected cases in the certain month of each year from 2005 to 2020 divided by the population.

The CISDCP is a national-level system that collects and manages data on infectious diseases and other health-related topics in China [10]. The system has been in place since the 1980s and has undergone significant improvements over the years to enhance its data quality and timeliness [15]. After the 2003 SARS epidemic, the updated CISDCP was also formally launched and provided a real-time and direct online reporting platform through the internet to monitor public health emergencies [10]. First-diagnosis doctors and laboratory personnel are responsible for the record and report by means of report cards and the internet [18]. With several procedures, such as amendments, supplementary reports, and reexamination, the data for all of the notifiable infectious diseases are summarized by the national CDC and released on the website of the Public Health Science Data Center [18]. In fact, reports of notifiable diseases from hospitals in real time have reduced the underreporting of infectious diseases. A monitoring report management system, personnel, and funding support ensure the system’s effective and sustainable operation [14].

Considering the comparability and continuity of data, we excluded the new and eradicated diseases such as SARS, Asian lineage avian influenza A, COVID-19, hepatitis D, and highly pathogenic avian influenza. Finally, 27 infectious diseases (2 class A notifiable infectious diseases and 25 class B notifiable infectious diseases in China) were included in the study. For our assessment, we categorized these 27 selected infectious diseases, including their subtypes, into 8 major types (quarantinable diseases, vaccine-preventable diseases, gastrointestinal diseases, vector-borne diseases, zoonotic infections, bacterial infections, sexually transmitted infections, and viral hepatitis) based on their characteristics and origins, according to Zhang and Wilson’s [15] categorization of notifiable infectious diseases in China (Table S3 in Multimedia Appendix 1).

### Statistical Analysis

Broken-line charts were used to visually illustrate the temporal trends of the yearly incidence of each type of disease from 2005 to 2020. We used 2 nonparametric methods, the Mann-Kendall test and Sen’s slope estimator (Technical Notes S1 and S2 in Multimedia Appendix 1), to determine whether there was a positive or negative trend in disease incidence and the corresponding statistical significance. The Mann-Kendall test is used to detect trends in time-series data, and it is based on the calculation of the Kendall rank correlation coefficient, which measures the strength and direction of monotonic trends [19]. By contrast, Sen’s method involves calculating the median slope of all possible pairs of data points in a time series, and it is more robust than other linear regression methods when dealing with outliers and nonnormal data [20].

We standardized the average monthly incidence rate of each infectious disease [0,1] based on percentile ranks and presented the standardized data in heat diagrams to illustrate seasonal distribution. Circular distribution analysis was used to test the seasonal trends of incidence and their statistical significance (Technical Note S3 in Multimedia Appendix 1). The seasonal peak reflects the concentration of cases in the time distribution. The peak day and peak period of the specific infectious diseases were calculated using this method.

Local Moran *I* statistic (Technical Note S4 in Multimedia Appendix 1) is a measure of spatial autocorrelation that evaluates whether neighboring observations in a data set are similar or dissimilar [21]. It can be used to identify spatial clusters, examine spatial dependence, and assess the impact of spatial outliers [16]. In this study, local Moran *I* was used to identify spatial clusters (ie, units with statistical significance) of infectious diseases, which can be further divided into hotspots (high-high clusters), coldspots (low-low clusters), and spatial outliers (low-high cluster and high-low clusters) [22]. Descriptive maps presenting the annual average incidence and spatial cluster maps highlighting these 4 types of clusters were created to depict the distribution of infectious disease cases. Additionally, 4 periods (2005-2008, 2009-2012, 2013-2016, and 2017-2020) were selected to create maps to visualize the spatiotemporal dynamics of infectious disease distribution. The incidence of each type of disease was classified into 4 classes using quantile classification and displayed in different colors on the descriptive maps. The class breakdown was calculated by taking the quartile of the values of all periods.

We used R 4.0.5 software (R Foundation) for the Mann-Kendall test and Sen’s scope estimates. Furthermore, Microsoft Excel 2019 was used to perform the circular distribution analysis, Moran *I* was computed using GeoDa 16.0 (version 1.18.0; GitHub, Inc), and the maps were created using ArcGIS 10.3 (version 10.0; Esri, Inc).

### Ethics Approval and Consent to Participate

This study only used secondary data, which are not applicable for the ethics committee approval.

## Results

Between January 1, 2005, and December 31, 2020, a total of 51,028,733 cases of 8 major types of infectious diseases were reported, among which 261,851 deaths were recorded (Table 1). The yearly average mortality was calculated as 120.584 deaths per 10,000,000 and the case-fatality ratio was 5.131 deaths per 1000 cases per year. Among the 8 types of infectious diseases, viral hepatitis (including A, B, C, E, and unspecified hepatitis) had the highest average yearly incidence (96.217/100,000; range 81.105-108.441), and sexually transmitted infections (AIDS, gonorrhea, and syphilis) resulted in the highest average yearly mortality (78.781/10,000,000; range 10.120-150.360).

**Figure 1:**
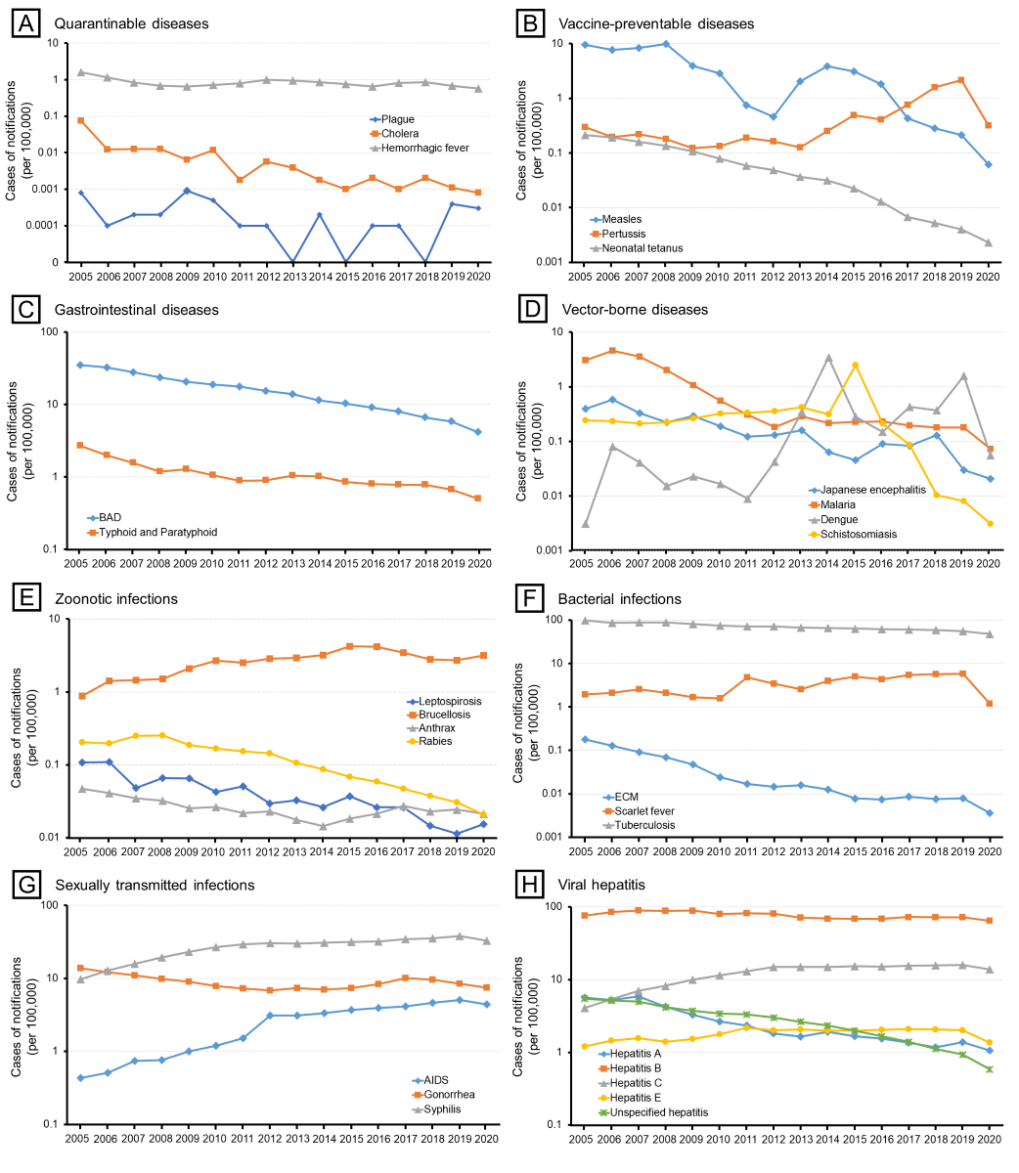
Incidence of 27 notifiable infectious diseases in China by category of disease, 2005-2020. BAD: bacillary and amoebic dysentery; ECM: epidemic cerebrospinal meningitis.

**Figure 2:**
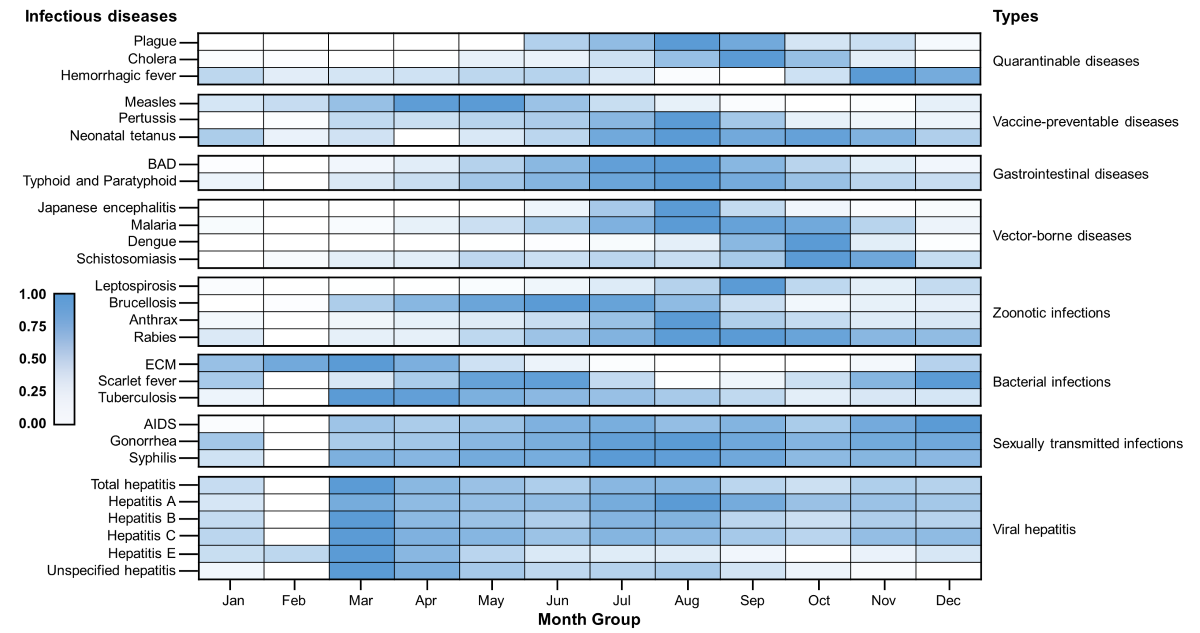
Incidence of 27 notifiable infectious diseases, by month, 2005-2020. BAD: bacillary and amoebic dysentery; ECM: epidemic cerebrospinal meningitis.

The incidence of 15 of the 27 infectious diseases significantly decreased during the research period, while 8 (pertussis, dengue fever, brucellosis, scarlet fever, AIDS, syphilis, and hepatitis C and E) of the 27 infectious diseases exhibited significantly increasing trends in incidence from 2005 to 2020 (Figure 1; detailed parameters are listed in Table 2). Syphilis, hepatitis C, and AIDS showed the fastest growth rates, with estimated slopes of 1.621, 0.759, and 0.327 annual new cases per 100,000, respectively. The incidences of plague, hemorrhagic fever, schistosomiasis, and gonorrhea were relatively stable. Six were characterized by significant seasonal distribution (Figure 2; detailed parameters are presented in Table S2 in Multimedia Appendix 1), namely, measles (incidence more concentrated from February 12 to July 8), bacillary and amoebic dysentery (BAD; May 9 to October 21), malaria (June 9 to November 8), dengue fever (February 26 to May 1), brucellosis (March 12 to September 15), and tuberculosis (January 7 to October 7).

**Table 1:** Incidence and mortality data for the 8 types of notifiable infectious diseases in China, 2005-2020.^a^

Type and infectious disease		Incidence data for infectious diseases				Mortality data for infectious diseases			Case-fatality ratios (per 1000)
		Cases (n)	Yearly incidence (per 100,000), (lower-upper limits)	Percentages of total cases	Deaths (n)		Yearly mortality (per 10,000,000), (lower-upper limits)	Percentages of total deaths	
Total		51,028,733	236.678 (184.204 to 272.388)	100.000	261,851		120.584 (75.870 to 178.860)	100.000	5.131
**Quarantinable diseases**		184,920	0.859 (0.580 to 1.682)	0.362	1716		0.802 (0.330 to 2.130)	0.655	9.280
	Plague	50	0 (0 to 0.001)	0.000	21		0.010 (0 to 0.020)	0.008	420.000
	Cholera	1991	0.009 (0.001 to 0.075)	0.004	7		0.004 (0 to 0.030)	0.003	3.516
	Hemorrhagic fever	182,879	0.850 (0.579 to 1.606)	0.358	1688		0.788 (0.320 to 2.080)	0.645	9.230
**Vaccine-preventable diseases**		849,924	3.992 (0.382 to 10.264)	1.666	1860		0.878 (0.020 to 2.790)	0.710	2.188
	Measles	731,114	3.448 (0.061 to 9.948)	1.433	450		0.221 (0 to 0.770)	0.172	0.615
	Pertussis	104,046	0.474 (0.121 to 2.150)	0.204	24		0.011 (0 to 0.030)	0.009	0.231
	Neonatal tetanus	14,764	0.070 (0.002 to 0.212)	0.029	1386		0.656 (0.010 to 2.350)	0.529	93.877
**Gastrointestinal diseases**		3,713,089	17.401 (4.618 to 37.791)	7.276	595		0.356 (0.010 to 1.230)	0.227	0.160
	Bacillary and amebic dysentery	3,473,204	16.280 (4.119 to 35.122)	6.806	520		0.247 (0.010 to 1.050)	0.199	0.150
	Typhoid and paratyphoid	239,885	1.120 (0.499 to 2.669)	0.470	75		0.036 (0 to 0.130)	0.029	0.313
**Vector-borne diseases**		433,171	2.027 (0.152 to 5.503)	0.849	2156		1.013 (0.100 to 3.820)	0.823	4.977
	Japanese encephalitis	38,435	0.181 (0.021 to 0.585)	0.075	1827		0.859 (0.060 to 3.540)	0.698	47.535
	Malaria	222,721	1.056 (0.073 to 4.604)	0.436	301		0.141 (0.040 to 0.350)	0.115	1.351
	Dengue fever	94,478	0.432 (0.003 to 3.458)	0.185	13		0.006 (0 to 0.040)	0.005	0.138
	Schistosomiasis	77,537	0.359 (0.003 to 2.506)	0.152	15		0.008 (0 to 0.030)	0.006	0.193
**Zoonotic infections**		642,425	2.963 (1.762 to 4.346)	1.259	23,739		11.158 (1.400 to 25.380)	9.066	36.952
	Leptospirosis	8276	0.039 (0.011 to 0.109)	0.016	168		0.080 (0 to 0.350)	0.064	20.300
	Brucellosis	604,789	2.786 (1.417 to 4.223)	1.185	14		0.007 (0 to 0.030)	0.005	0.023
	Anthrax	5204	0.024 (0.014 to 0.041)	0.010	53		0.024 (0 to 0.090)	0.020	10.184
	Rabies	24,156	0.114 (0.014 to 0.251)	0.047	23,504		11.047 (1.340 to 25.110)	8.976	973.009
**Bacterial infections**		16,035,563	74.497 (48.948 to 98.986)	31.425	46,794		21.731 (13.700 to 29.040)	17.870	2.918
	Epidemic cerebrospinal meningitis	8453	0.040 (0.004 to 0.178)	0.017	845		0.400 (0.020 to 1.580)	0.323	99.965
	Scarlet fever	734,758	3.381 (1.180 to 5.853)	1.440	10		0.005 (0 to 0.020)	0.004	0.014
	Tuberculosis	15,292,352	71.076 (47.764 to 96.879)	29.968	45,939		21.326 (13.670 to 28.490)	17.544	3.004
**Sexually transmitted infections**		8,406,048	38.722 (24.032 to 51.911)	16.473	172,249		78.781 (10.120 to 150.360)	65.781	20.491
	AIDS	570,576	2.605 (0.432 to 5.099)	1.118	171,240		78.311 (5.700 to 150.360)	65.396	300.118
	Gonorrhea	1,936,211	8.987 (6.817 to 13.872)	3.794	15		0.008 (0 to 0.020)	0.006	0.008
	Syphilis	5,899,261	27.130 (9.727 to 38.368)	11.561	994		0.463 (0.280 to 0.660)	0.380	0.168
**Viral hepatitis**		20,763,593	96.217 (81.105 to 108.441)	40.690	12,742		5.939 (3.470 to 10.330)	4.866	0.614
	Hepatitis A	571,552	2.678 (1.055 to 5.868)	1.120	200		0.094 (0.010 to 0.330)	0.076	0.350
	Hepatitis B	16,533,416	76.659 (64.286 to 89.005)	32.400	9704		4.524 (2.580 to 7.610)	3.706	0.587
	Hepatitis C	2,656,625	12.219 (4.072 to 16.015)	5.206	1897		0.879 (0.700 to 1.220)	0.724	0.714
	Hepatitis E	388,867	1.796 (1.196 to 2.178)	0.762	423		0.197 (0.080 to 0.410)	0.162	1.088
	Unspecified hepatitis	611,639	2.865 (0.584 to 5.481)	1.199	518		0.244 (0.010 to 0.890)	0.19	0.847

^a^Incidence data and mortality data for infectious diseases are from January 2005 to December 2020. Incidence was calculated as the number of annual cases divided by the population size; mortality as the number of deaths per year divided by the population size; and the case-fatality ratio as the number of annual deaths divided by the number of annual cases. The specific incidence and mortality rates for each type of disease were also calculated. Yearly incidence and yearly mortality are averages for notifiable periods.

**Table 2:** Time and seasonal trends of 8 types of notifiable infectious diseases in China, 2005-2020.

Type and infectious disease		Time trend^a^			Seasonal trend^b^	
		Mann-Kendall trend	*P* value	Sen’s slope	Seasonal feature^c^	*P* value
Total		Decrease	<.001	–4.735	July 14 (April 13 to October 13)	<.001
**Quarantinable diseases**						
	Plague	Stable	.25	–0.000	Not significant	.99
	Cholera	Decrease	<.001	–0.000	Not significant	.92
	Hemorrhagic fever	Stable	.10	–0.021	Not significant	.73
**Vaccine-preventable diseases**						
	Measles	Decrease	<.001	–0.616	April 25 (February 12 to July 8)	<.001
	Pertussis	Increase	.03	0.028	Not significant	.70
	Neonatal tetanus	Decrease	<.001	–0.013	Not significant	.99
**Gastrointestinal diseases**						
	Bacillary and amoebic dysentery	Decrease	<.001	–1.751	July 30 (May 9 to October 21)	<.001
	Typhoid and paratyphoid	Decrease	<.001	–0.072	Not significant	.47
**Vector-borne diseases**						
	Japanese encephalitis	Decrease	<.001	–0.025	Not significant	.12
	Malaria	Decrease	<.001	–0.131	August 24 (June 9 to November 8)	.04
	Dengue fever	Increase	.01	0.025	March 30 (February 26 to May 1)	.006
	Schistosomiasis	Stable	.26	–0.013	Not significant	.56
**Zoonotic infections**						
	Leptospirosis	Decrease	<.001	–0.004	Not significant	.78
	Brucellosis	Increase	.001	0.130	June 13 (March 12 to September 15)	.03
	Anthrax	Decrease	.01	–0.001	Not significant	.95
	Rabies	Decrease	<.001	–0.015	Not significant	.96
**Bacterial infections**						
	Epidemic cerebrospinal meningitis	Decrease	<.001	–0.006	Not significant	.84
	Scarlet fever	Increase	.02	0.276	Not significant	.86
	Tuberculosis	Decrease	<.001	–2.733	May 23 (January 7 to October 7)	.003
**Sexually transmitted infections**						
	AIDS	Increase	<.001	0.327	Not significant	.68
	Gonorrhea	Stable	.10	–0.250	Not significant	.65
	Syphilis	Increase	<.001	1.621	Not significant	.20
**Viral hepatitis**						
	Hepatitis A	Decrease	<.001	–0.287	Not significant	.90
	Hepatitis B	Decrease	.003	–1.400	Not significant	.47
	Hepatitis C	Increase	<.001	0.759	Not significant	.90
	Hepatitis E	Increase	.04	0.046	Not significant	.66
	Unspecified hepatitis	Decrease	<.001	–0.317	Not significant	.91

^a^The nonparametric Mann-Kendall trend test and Sen’s methods were applied to determine the magnitude and significance of the time trends. The estimated slope indicates the number of yearly new cases per 100,000 during the study period.

^b^The circular distribution method was applied to analyze the seasonal feature and significance. The seasonal peak reflects the concentration of cases in the time distribution, while the period in parentheses indicates a significant seasonal feature, which is the peak period of the specific infectious diseases. The incidence is more concentrated during this period. The date outside the parentheses indicates the peak day, where the incidence is speculated to reach the highest.

^c^Data presented as month date.

**Figure 3:**
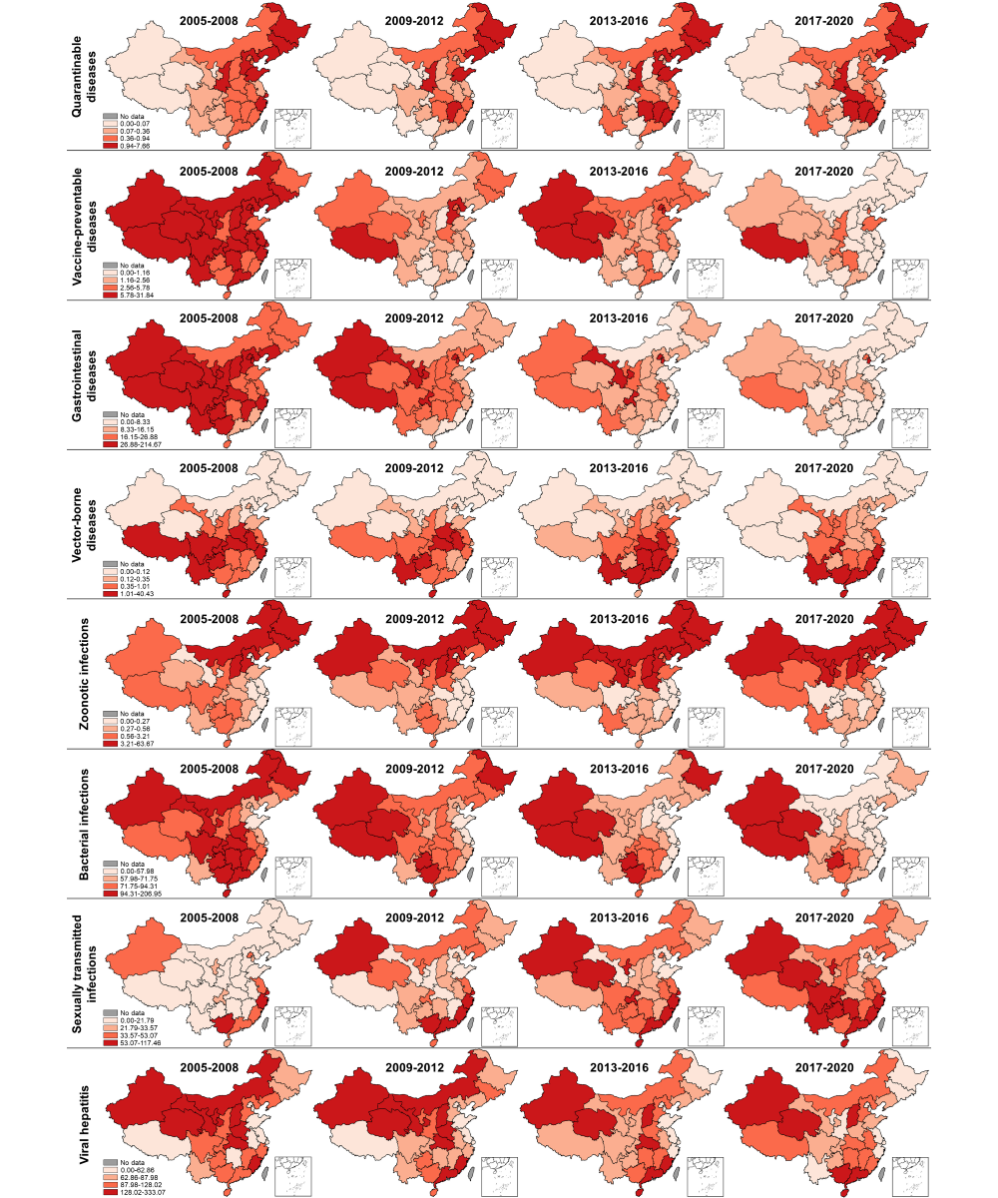
Descriptive maps of 8 types of notifiable infectious diseases in 2005-2008, 2009-2012, 2013-2016, and 2017-2020.

**Figure 4:**
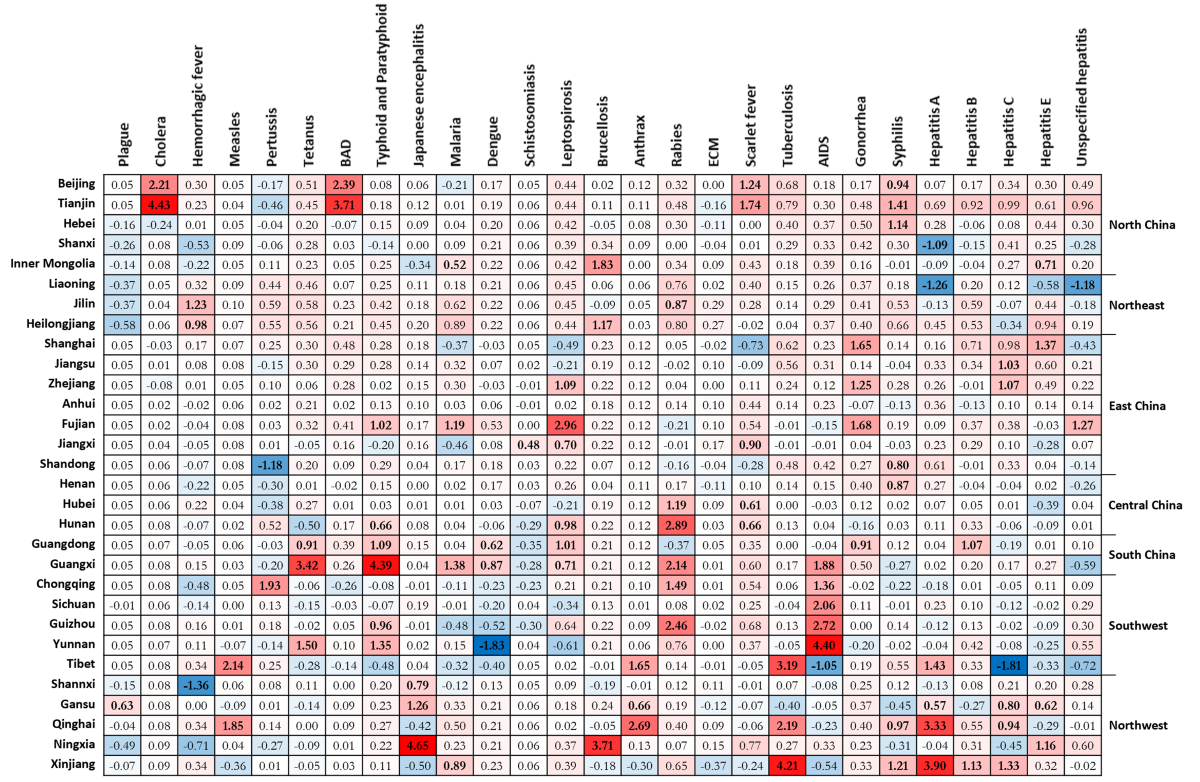
Local Moran *I* of 27 notifiable infectious diseases at the provincial level in 2017-2020. BAD: bacillary and amoebic dysentery; ECM: epidemic cerebrospinal meningitis.

**Figure 5:**
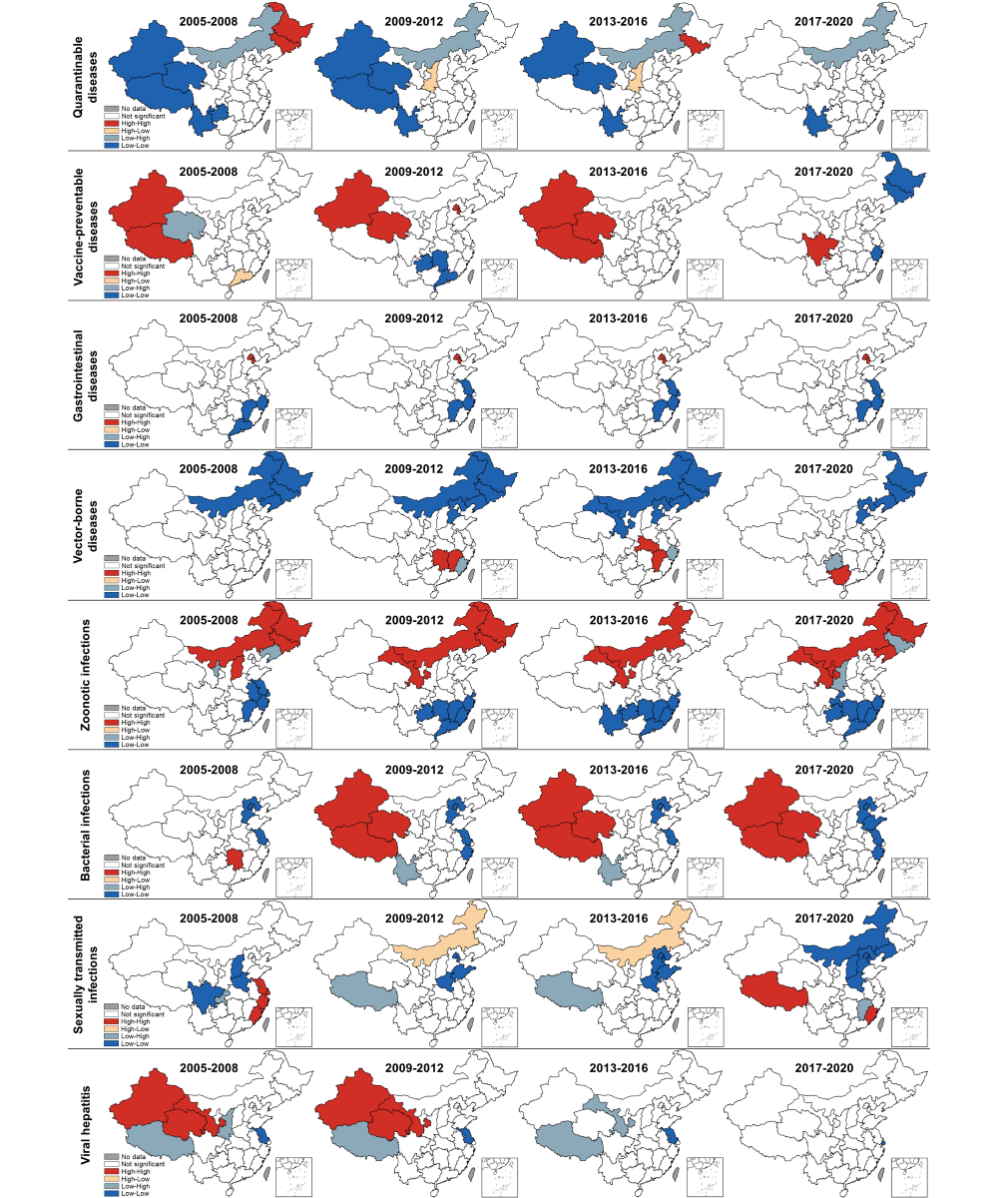
Spatial cluster maps of 8 types of notifiable infectious diseases in 2005-2008, 2009-2012, 2013-2016, and 2017-2020.

Quarantinable diseases, vaccine-preventable diseases, gastrointestinal diseases, and vector-borne diseases showed a gradually improving situation (Figure 3), with the incidence declining in almost all the provinces (red color becoming lighter on maps). By contrast, the geographical range with a high incidence of zoonotic infections expanded from North China (Inner Mongolia and Shanxi) to Northwest (Xinjiang and Ningxia); bacterial infections followed a general trend in which they become more severe in inland provinces (Northwest) over time, and the coastal provinces generally reported a decreasing incidence; and the geographical range with a high incidence of sexually transmitted infections rapidly expanded to almost the whole country. Global Moran *I* revealed a concentration tendency of high-incidence units for most types of infectious diseases (Table S4 in Multimedia Appendix 1) and local Moran *I* reflected the regional spatial correlation and clusters (Figures 4 and S1-S3 in Multimedia Appendix 1). During 2017-2020, as shown in Figure 5, no high-high cluster area was detected in the case of quarantinable diseases and viral hepatitis; furthermore, only 1 high-high cluster area each was detected in the case of vaccine-preventable diseases (Sichuan) and vector-borne diseases (Guangxi). By contrast, the high-high cluster areas of the other 4 groups of infectious diseases were concentrated in more provinces, including gastrointestinal diseases (Beijing and Tianjin), zoonotic infections (Heilongjiang, Inner Mongolia, Liaoning, Ningxia, and Gansu), bacterial infections (Xinjiang, Tibet, and Qinghai), and sexually transmitted infections (Tibet and Fujian). The descriptive and spatial cluster maps of 27 included disease are displayed in Figures S4-S30 in Multimedia Appendix 1.

## Discussion

### Principal Findings

We reported the latest spatiotemporal and seasonal trends of 8 types of notifiable infectious diseases in China. The results reveal that viral hepatitis, bacterial infections, and sexually transmitted infections are the 3 main groups of diseases among the cases reported between January 2005 and December 2020, accounting for 88.59% (45,205,204/51,028,733) of the cases attributed to the 27 selected infectious diseases. Furthermore, the incidence of 8 (pertussis, dengue fever, brucellosis, scarlet fever, AIDS, syphilis, and hepatitis C and E) of the 27 selected notifiable infectious diseases is still increasing in China. Measles (*P*<.001), BAD (*P*<.001), malaria (*P*=.04), dengue fever (*P*=.006), brucellosis (*P*=.03), and tuberculosis (*P*=.003) were found to be characterized by significant seasonal distribution, while AIDS and tuberculosis accounted for 82.94% (217,179/261,851) of the deaths associated with the 27 selected diseases. Disparities were observed in the burden of most infectious diseases, with a shift of burden of bacterial infections and sexually transmitted infections from coastal to inland provinces.

### Quarantinable Diseases

Outbreaks of plague and cholera, the deadliest infectious diseases in China and the world, have occurred since the early 19th century, and waves of infection continue to occur until today. One of the globally known combative programs, the Wu Lien-Teh, eradicated plague through a series of measures, such as the wearing of masks, initiating quarantine, and cremating plague victims, from 1910 to 1911 [23]. After the People’s Republic of China was founded, the number of annual plague cases decreased dramatically to less than 100 cases per year, which persisted until the end of the 1990s. By contrast, 3 major outbreaks of cholera with more than 20,000 cases were recorded from 1950 to 2000 [24]. Since the beginning of the 21st century, plague and cholera, the only 2 class A notifiable infectious diseases in China, persist at very low endemic levels with a continuously decreasing incidence, which may be attributed to improved health-related awareness and strict control measures. Our study demonstrates that plague and cholera were well-tamed, while hemorrhagic fever remained active across all provinces in China. Overall, there were 50 plague cases, 1991 cholera cases, and 182,879 hemorrhagic fever cases between 2005 and 2020 (Table 1). By contrast, the incidence of hemorrhagic fever remained relatively stable after 2010 (Figure 1A) and it was highly variable across different regions, while the disparities in the burden have gradually reduced, with provinces in Northeast China identified as high-risk areas for hemorrhagic fever (Figure S6 in Multimedia Appendix 1). In China, Hantaan virus, Seoul virus, and Puumala virus were identified as the predominant serotypes of hantavirus [25,26].

### Vaccine-Preventable Diseases

Our study reveals that most vaccine-preventable diseases in China are under control despite seasonal, sporadic outbreaks of measles and pertussis in the country. The continuous efforts in increasing vaccine coverage play an important role in combating vaccine-preventable diseases [27]. In the 1960s, a combined diphtheria-tetanus-pertussis vaccine and 3 attenuated measles vaccine strains (Beijing-55, Shanghai-191, and Changchun-47) were developed and introduced [28]. Moreover, in 1978, China implemented the first expanding strategy for the National Immunization Program based on WHO guidelines [29]. In 1982, the Ministry of Health set vaccine coverage targets of 80%-90% for diphtheria-tetanus-pertussis and 90%-95% for measles by school age [30]. Since 2005, citizens have been vaccinated for measles, pertussis, and tetanus for free, as both vaccines are included in the National Immunization Program, which is fully government funded [31]. From 2005 to 2020, measles accounted for more than 86.02% (731,114/849,924) of the total cases of vaccine-preventable diseases, with Tibet and Qinghai identified as a high-high cluster area during 2017-2020 (Figure S7 in Multimedia Appendix 1). There was a resurgence of measles in 2013-2016 with a seasonal peak in April, indicating that many children were not vaccinated in time [32]. Among the 3 selected vaccine-preventable diseases, pertussis was the only one that exhibited a significant increase in incidence (measles and neonatal tetanus significantly declined), with a total of 104,046 cases reported during the study period. During 2017-2020, pertussis cases were still detected across several regions, with Chongqing identified as a high-high cluster area (Figure S8 in Multimedia Appendix 1). Recent studies have found that a substantial proportion of the population is susceptible to pertussis due to vaccination and regional discrepancies in protective antibody rates, suggesting the need for introducing a booster to protect children [33,34]. By contrast, the incidence of neonatal tetanus continued to decrease in almost all the provincial units (Figure S9 in Multimedia Appendix 1).

### Gastrointestinal Diseases

In the 20th century, the number of reported cases of gastrointestinal diseases continued to decline due to improvements in sanitary conditions, along with the development of the live oral Shigella vaccine and Vi polysaccharide vaccine [35,36]. Our study results reveal an endemic situation and geographical disparity regarding gastrointestinal diseases, with a higher burden identified in the densely populated Beijing-Tianjin-Hebei Region (BAD; Figure S10 in Multimedia Appendix 1) and Southwest China (typhoid and paratyphoid fever; Figure S11 in Multimedia Appendix 1). However, a previous study that focused on the Beijing-Tianjin-Hebei region found bacillary dysentery to pose a higher risk on children [37]. A possible reason for the higher typhoid and paratyphoid fever incidence in Southwest China is that it borders Southeast Asia (Vietnam, Laos, and Myanmar), which has a high-risk population due to poor sanitation and unsafe food and water [38]. In addition, the residents of Southwest China have a long history of typhoid and paratyphoid fever [39].

### Vector-Borne Diseases

Our study clearly shows China’s great achievements in controlling vector-borne diseases and highlights its urgency in meeting the challenges posed by dengue fever. After nearly 70 years of battling malaria (since 1949), a milestone of 0 indigenous malaria cases was achieved in 2017 [40,41]. In June 2021, China was officially awarded a malaria-free certification by the WHO, making it the first country in the WHO Western Pacific Region to be certified in more than 3 decades [42]. During our research period, there were only a few small-scale regional outbreaks of malaria in Yunnan and Anhui provinces (Figure S13 in Multimedia Appendix 1). The number of Japanese encephalitis and schistosomiasis cases both decreased dramatically to less than 0.01 cases per 100,000 in 2020, with only controllable risks in local areas. Japanese encephalitis is a typical seasonal epidemic disease with an 80-year recorded history in China, and approximately 90% of cases are reported from July to September. The first Japanese encephalitis case was reported in 1949, and there were 2 devastating epidemics in the 1960s and 1970s. Subsequently, the number of cases decreased dramatically due to the use of commercial vaccines in the 1980s, which were then included in China’s National Immunisation Program [30]. Unsurprisingly, high-high cluster areas of Japanese encephalitis cases were detected in southwest China, which has high temperatures, rainfall, and humidity; however, this transferred to Northwest China (Shaanxi, Gansu, and Ningxia) during 2017-2020 (Figure S12 in Multimedia Appendix 1). Schistosomiasis, one of the oldest known infectious diseases in China, has persisted for over 2 millennia according to corresponding historical records [43]. Large-scale schistosomiasis control measures have been implemented in China since the mid-1950s; however, there were still 240 counties in 8 provinces affected by schistosomiasis in 1989 [44]. Long-term risks of schistosomiasis transmission still exist in local areas, with high-high clusters identified in Anhui, Hunan, and Jiangxi in recent years (Figure S15 in Multimedia Appendix 1). Spatial analysis at smaller scales suggests that the high-risk areas are detected near the Yangtze River, the Poyang Lake region, and the Dongting Lake region [45]. Furthermore, some provinces are faced with an increasingly severe threat of imported schistosomiasis from Africa [43]. Great success in controlling vector-borne diseases cannot be achieved without strong political commitment, strengthening of health systems, timely reporting, and the evolution of control strategies and technologies [44]. By contrast, dengue fever was the only vector-borne disease with a continuously increasing disease burden; additionally, it is the most prevalent mosquito-borne viral disease affecting people worldwide [46]. High-risk areas were found in southern provinces due to outbreaks in Guangdong, Yunnan, and Fujian in recent years (Figure S14 in Multimedia Appendix 1) [47]. These provinces are adjacent to Southeast Asia and share an integrated network of economies with Southeast Asian countries, boosting immigration and contributing to the transmission of tropical diseases such as dengue fever. In the existing literature, there are debates as to whether dengue fever is an imported disease in China [48,49], with the possibility of localization being recognized.

### Zoonotic Infections

Our study exhibits the evolution of brucellosis from a sporadic disease to a major public health concern due to an increase in its geographical range and the number of cases. During the 21st century, brucellosis was the only 1 among the 4 selected zoonotic infections with increasing incidence and the only 1 characterized by significant seasonality. In 2020, 47,245 diagnosed cases were reported in China, with an incidence of 3.37 cases per 100,000. This disease tends to occur predominantly from March to September (Table 2). By contrast, the incidence of leptospirosis, human rabies, and anthrax diminished to less than 0.03 cases per 100,000 in 2020, which is possibly due to the country’s investment in health worker training and increased access to postexposure prophylaxis [50,51]. Historically, human brucellosis has primarily affected northern China; however, there have been increasing reports of outbreaks in other regions in recent years. High-risk regions were found in provinces with highly developed animal husbandry such as Inner Mongolia, Gansu, Ningxia, Liaoning, and Heilongjiang (Figure S17 in Multimedia Appendix 1); in fact, spatial analysis at smaller scales indicates that high-risk areas overlap with high-livestock-density areas, especially those with high densities of sheep and goats [52,53]. The high-risk areas for leptospirosis were detected in southern China (Figure S16 in Multimedia Appendix 1), which could be attributed to both environmental and socioeconomic factors [54]. By contrast, the high-risk areas of anthrax were found in western China (Figure S18 in Multimedia Appendix 1) and the high-risk areas of rabies were concentrated in southern China (Figure S19 in Multimedia Appendix 1).

### Bacterial Infections

Our study demonstrates the quick shift of tuberculosis burden from coastal to inland provinces and the resurgence of scarlet fever after 2010. During the study period, tuberculosis was the leading cause of death apart from AIDS, although there was a slight decrease in the incidence of tuberculosis from 96.88 per 100,000 to 47.76 per 100,000. High-high clusters of tuberculosis were mainly distributed in the western region due to the underdeveloped economy and poor medical resources, as well as in southwest China due to rainfall and humidity (Figure S22 in Multimedia Appendix 1) [18]. Notably, the spring-summer predominance of tuberculosis was detected in China and other countries worldwide, which is different from the recognized winter season for respiratory infections [55]. Several hypotheses for its spring-summer surge have been proposed, including a dominant theory of the indoor congregation in wintertime or seasonal variation in meteorological factors or both [56]. A recent study in Hong Kong showed that the pattern is largely contributed to by reactivation diseases precipitated by defective immunity [57]. In comparison, the incidence of scarlet fever remained stable from 2005 to 2010; subsequently, there was a remarkable increase to nearly 5 cases per 100,000 in 2011, after which it remained steady. The average incidence after the 2011 upsurge of scarlet fever was approximately 2 times the average incidence from 2005 to 2010. New cases were mainly found in northern China (Figure S21 in Multimedia Appendix 1). However, no significant seasonal characteristics were detected in the national trends of the incidence of scarlet fever, although a subregional analysis revealed semiannual seasonal patterns (May-June and November-December) in the north and south of China, while Southwest China had only 1 peak (May-June) [58]. By contrast, the high-high cluster of epidemic cerebrospinal meningitis was only detected in Tibet during 2017-2020 (Figure S20 in Multimedia Appendix 1).

### Sexually Transmitted Infections

Our study reveals an increasing trend in AIDS and syphilis epidemics, accompanied by significant spatial disparities in the burden of all sexually transmitted infections. We found that AIDS caused a larger number of deaths and is particularly prominent in the southwestern area (Figure S23 in Multimedia Appendix 1). One possible reason is the geographical proximity of these regions to Southeast Asia, which is known for its drug and prostitution industry [59]. The first case of AIDS was discovered in Yunnan (a southwest border province) in 1985 [60]. Since then, HIV has spread quickly from drug users and blood transfusion recipients to others, and the HIV upsurge in young people through mainly sexual routes is particularly concerning [61]. During our study period, a larger number of syphilis and gonorrhea cases were found in the Yangtze River Delta region (Figure S24 in Multimedia Appendix 1). This is not surprising given that it is 1 of the 3 most integrated and dynamic urban regions, consisting of 4 provinces and several highly developed cities [62]. However, the high-high clusters within these provinces gradually disappeared, and newly diagnosed syphilis cases were reported more frequently in western provinces (Figure S25 in Multimedia Appendix 1). This is likely a result of the limited diagnostic capacity in these less-developed provinces, resulting in the continued spread [63]. Sexually transmitted infections are highly private and the cultural atmosphere in China is rather conservative; therefore, knowledge promotion regarding sexually transmitted infections is restricted to sex education in communities, while the rapid urbanization, growing mobility of the population, and changes in sexual behaviors increase the difficulty of sexually transmitted infection prevention and control [64]. In the Chinese context, screening the most-at-risk populations remains the most effective strategy for early detection and timely treatment [62]. In fact, prevention of mother-to-child transmission and antenatal care screening among pregnant women are believed to be the most successful screening strategies for HIV and syphilis, and particular attention should be focused on gonorrhea in men who have sex with men [60].

### Viral Hepatitis

Our study demonstrates the distinct epidemiologic trends of fecal-oral transmitted hepatitis (A and E) and blood-borne hepatitis (B and C), with the latter resulting in a significant burden on communities. The fecal-oral transmitted hepatitis A and E exhibited opposite trends and inconsistent spatial cluster features. Notably, the incidence of hepatitis A declined from 2005 to 2020, which may be attributed to the improved sanitation and hygiene conditions in China. This also explains why the hotspots were located in the undeveloped western units (Xinjiang, Tibet, Qinghai, Gansu, etc; Figure S26 in Multimedia Appendix 1). However, it appears that hepatitis E was not controlled well, as it exhibited an upward trend from 2005 to 2011 and has remained relatively stable until today (Table 2). With hotspots located in the Yangtze River Delta region, hepatitis E infections usually occur as sporadic cases (Figure S29 in Multimedia Appendix 1). Furthermore, the blood-borne hepatitis B and C differed in epidemiological trends and high-risk areas. Despite the progress made in the implementation of hepatitis B virus vaccine programs [65-67], the status of hepatitis B remained relatively stable during the study period, and hotspots were newly detected in Guangdong during 2017-2020 (Figure S27 in Multimedia Appendix 1). It is believed that the vaccination-for-free strategy targeting only children under 14 years old resulted in adults’ low active hepatitis B vaccination rate [68], and mother-to-child transmission still poses a challenge [69]. In 2005, the incidence of hepatitis C was quite low (4.07/100,000), while it exceeded 13.82/100,000 in 2020, with Gansu identified as a hotspot (Figure S28 in Multimedia Appendix 1). It is believed that hepatitis C virus infection has not received due attention in China, as many people are unaware of their infections, which potentially accelerates its transmission [21]. In particular, hepatitis B virus, hepatitis C virus, and HIV can all be transmitted through the exchange of body fluids, thus sharing similar most-at-risk populations in China, including drug users and men who have sex with men [70,71]. Moreover, the high-high cluster area of unspecified hepatitis was detected in southeast China (Figure S30 in Multimedia Appendix 1).

### Limitations

Our study had some limitations. First, as an ecological study, there is a risk of ecological fallacy, where trends in the geographical resolution we used might not have represented true subnational trends. Specifically, causality at the individual level could not be inferred based on a higher incidence in a geographical unit. Second, due to data availability–related issues, this study only investigated the spatiotemporal distribution patterns of notifiable infectious disease cases at the provincial level; however, smaller spatial scales (such as the city or county level) contribute exponentially to the applicability and significance of the analysis. Third, the national surveillance system is not an exhaustive source and is potentially systematically biased in some geographical units. Furthermore, there are differences in screening and reporting capacity between coastal and inland provinces due to different economic development levels; therefore, some cases were not included as the patients did not seek medical services, and the reported yearly incidence could have been underestimated in some provinces. The surveillance system may also suffer delays during Chinese New Year (usually in January or February). This explains the sudden surge of cases in March for many diseases. In the year 2020, the incidence of many types of notifiable infectious diseases decreased. We believe that the decreasing trend may be overestimated as the nationwide nonpharmaceutical interventions and public risk perception in the COVID-19 epidemic may have had a broad impact on infectious disease notification and follow-up examinations [7,72]. Another limitation of this study is that we only included 27 notifiable infectious diseases to facilitate the comparison of results and we did not examine bivariate or multivariate relationships among these 27 diseases or diseases with similar epidemiological causes. Therefore, further studies are required to examine disease comorbidity and the cost-effectiveness of disease-combined interventions.

### Conclusions

Some types of infectious diseases, especially viral hepatitis, bacterial infections, and sexually transmitted infections, continue to impose a significant burden on the population in China. Significant disparities were observed in the burden of most infectious diseases, with a shift from coastal to inland provincial units. Therefore, prevention and control policies that vary across localities should be formulated to target the identified up-to-date high-risk areas.

## Appendix

Multimedia Appendix 1 Supplementary Materials: Technical Note, Additional Figures and Tables.

## Abbreviations

BAD: bacillary and amoebic dysentery
CDC: Centers for Disease Control and Prevention
CISDCP: China Information System for Disease Control and Prevention
WHO: World Health Organization

## Data Availability

Additional data are available upon request from the corresponding author.
