# Supplementary material for: Spatiotemporal and Seasonal Trends of Class A and B Notifiable Infectious Diseases in China: Retrospective Analysis
Source: JMIR Public Health Surveill. 2023 Apr 27;9:e42820. doi: 10.2196/42820 (PMC10176137; doi:10.2196/42820)

**Supplementary Materials: Technical Note, Additional Figures and Tables**

**Technical Note S1. Formulas of Mann-Kendall test statistic**

The Mann–Kendall statistical test has been frequently used to quantify the significance of trends in hydrometeorological time series. The Mann-Kendall test statistic $S$ is calculated as

$$S=\sum_{i=1}^{n-1} \sum_{j=i+1}^{n} sgn(y_{j}-y_{i})$$

Where $n$ is the number of data points included in the analysis, $x_{i}$ and $x_{j}$ are the yearly incidence in the $i$th year and the $j$th year ($j$>$i$), and $sgn(x_{j}-x_{i})$ is the sign function as:

$$sgn\left( x_{j}-x_{i} \right)=\left\{ \begin{aligned} +1, if x_{j}-x_{i}>0 \\ 0, if x_{j}-x_{i}=0 \\ -1, if x_{j}-x_{i}<0 \end{aligned} \right.$$

The variance is computed as

$$Var(S)=\frac{n\left( n-1 \right)\left( 2n+5 \right)-\sum_{i=1}^{m} t_{i}(t_{i}-1)(2t_{i}+5)}{18}$$

Where $n$ is the number of years included in the analysis, $m$ is the number of tied groups and $t_{i}$ denotes the number of data points in the $m$th group. A tied group is a set of sample data having the same value. As the number of years included in the analysis $n>10$, the standard normal test statistic $Z_{S}$ is computed using the following Formula:

$$Z_{S}=\left\{ \begin{aligned} \frac{S-1}{\sqrt{Var(S)}}, if S>0 \\ 0, if S=0 \\ \frac{S+1}{\sqrt{Var(S)}}, if S<0 \end{aligned} \right.$$

Based on the $Z_{S}$ statistics, we decided to accept or reject the null hypothesis H_0_. When the time trend is stable ($\left| Z_{S} \right|>Z_{1-\alpha/2}$), H_0_ is accepted. Otherwise, H_0_ is rejected. A significance level of 5% was adopted for this study. Positive values of $Z_{S}$ indicate increasing trends while negative $Z_{S}$ values show decreasing trends.

**Technical Note S2. Sen's slope estimator**

Sen's slope estimator is a non-parametric procedure to estimate the slope of trend in the sample of N pairs of data. Sen's method uses a linear model to estimate the slope of the trend, and the variance of the residuals should be constant in time.

$$Q_{k}=\frac{x_{j}-x_{i}}{j-i}, if k=1, 2, 3,\ldots, N$$

Where $x_{j}$ and $x_{i}$ are the yearly incidence in the $i$th year and the $j$th year ($j$>$i$) in this study. As well for this study, there was only one datum in each year included in the analysis, then $N=\frac{n(n-1)}{2}$, where $n$ was the number of years included in this study. The $N$ values of $Q_{k}$ are ranked from smallest to largest and the median of slope or Sen's slope estimator is computed as:

$$Q_{med}=\left\{ \begin{aligned} Q_{\left[ \left( N+1 \right)/2 \right]}, if N is odd \\ \frac{Q_{\left[ N/2 \right]}-Q_{\left[ \left( N+2 \right)/2 \right]}}{2}, if N is even \end{aligned} \right.$$

The $Q_{med}$ sign reflects yearly incidence trend reflection, while its value indicates the steepness of the trend. To determine whether the median slope is statistically different than zero, one should obtain the confidence interval of $Q_{med}$ at specific probability. The confidence interval about the time slope can be computed as follows:

$$C_{\alpha}=Z_{1-\alpha/2}\sqrt{\frac{n\left( n-1 \right)\left( 2n+5 \right)-\sum_{i=1}^{m} t_{i}(t_{i}-1)(2t_{i}+5)}{18}}$$

Where $Z_{1-\alpha/2}$ is obtained from the standard normal distribution table. In this study, a significance level of 5% was adopted. Then, $M_{1}=\frac{N-C_{\alpha}}{2}$ and $M_{2}=\frac{N+C_{\alpha}}{2}$ are computed. The lower limits $Q_{min}$ and upper limits $Q_{max}$ of the confidence interval are the $M_{1}$th largest and the ${(M}_{2}+1)$th largest of the $N$ ordered slope estimates. The slope $Q_{med}$ is statistically different than zero if the two limits ($Q_{min}$ and $Q_{max}$) have similar signs.

**Technical Note S3. Circular Distribution Analysis**

A circular statistical approach is used to test the seasonality and identify the high-risk times. The approach considers the date of incidence of infectious diseases within a year as polar coordinates on the circumference of a unit circle centered at the origin. Note that taking the median of each month as incidence date in this study, each date ($D_{i}$) thus corresponds to each direction $(\alpha_{i}$), and each direction $(\alpha_{i}$) corresponds to a point on the circumference of the unit circle. The angular position of the date of occurrence $D_{i}$ of the incidence each month is defined using:

$$\alpha_{i}=D_{i}\left( \frac{2\pi}{365} \right)$$

The $\bar{x}$ and $\bar{y}$ coordinates of the peak date of incidence rate around the year are determined using:

$$\bar{x}=(\sum f_{i}cos\alpha_{i})/n$$

$$\bar{y}=(\sum f_{i}sin\alpha_{i})/n$$

Where $f_{i}$ is the incidence in the $i$th month, and $n$ is the total incidence all over the years. The direction representing the peak date of incidence rate is then obtained using:

$$\bar{\alpha}={tan}^{-1}\left( \frac{\bar{y}}{\bar{x}} \right)$$

Mapping the direction $\bar{\alpha}$ to the date, the peak date of incidence rate around the year is obtained by:

$$\bar{D}=\bar{\alpha}\left( \frac{365}{2\pi} \right)$$

The variability of incidence of infectious diseases occurrences about the peak date is obtained using the mean resultant length:

$$\rho=\sqrt{\bar{x}^{2}+\bar{y}^{2}}$$

$\rho$ is a dimensionless measure of the spread of the data and the value of $\rho$ ranges from 0 (indicating greater variability) to 1 (indicating all the incidence occurred on the same day of the year). The circle standard deviation is calculated based on the following equation:

$$S=\sqrt{-2\mathrm{In}\rho}$$

Converting S to days $M=S\left( \frac{365}{2\pi} \right)$. As a result, the peak interval of incidence rate is [$\bar{D}-M, \bar{D}+M$]

The hypothesis test needs to calculate Rayleigh's Z:

$$Z=nr^{2}$$

When $Z>Z_{1-\alpha/2}$, we think there is a significant peak in the incidence rate. For this study, a significance level of 5% was adopted.

**Technical Note S4. Formulas of global and local Moran's I**

Two forms (global and local) of Moran's I statistic were adopted to explore the regional disparities in the burden of notifiable infectious diseases among the 31 provincial units. Moran's I is a widely accepted measure for describing the spatial distributions of disease cases and for identifying unusual units or subsets of units. Global Moran's I is a measure for describing the overall spatial distribution characteristic of the entire area, while local Moran's I is the decomposition of global Moran's I for a particular area. That is, the sum of local Moran's I is proportional to global Moran's I. Global Moran's I varies between −1 and 1, while local Moran's I has no value limit. A positive value of global Moran's I indicates the infectious disease cases are clustered on a map, whereas a negative value implies the infectious disease cases are dispersed on a map. The described tendencies are more significant when the value approaches −1 and 1, whereas 0 indicates that the infectious disease cases are randomly dispersed.

The formulas of global and local Moran's I are defined as:

$$global {Moran}^{'}s I=\frac{n\sum_{i=1}^{n} \sum_{j=1}^{n} W_{ij}\left( y_{i}-\bar{y} \right)\left( y_{j}-\bar{y} \right)}{\sum_{i=1}^{n} \sum_{j=1}^{n} W_{ij}\sum_{i=1}^{n} \left( y_{i}-\bar{y} \right)^{2}}$$

$${local Moran}^{'}s I=\frac{\left( y_{i}-\bar{y} \right)}{m_{0}}\sum_{j} W_{ij}\left( y_{j}-\bar{y} \right) m_{0}=\sum_{i} {\left( y_{i}-\bar{y} \right)^{2}}/n.$$

In the formula, n is the number of units in total, $y_{i}$ and $y_{j}$ are the incidence of a certain disease of units i and j, $\bar{y}$ is the average value. $W$ stands for the row-standardized spatial weight matrix, $W_{ij}$ is 1 if units i and j are adjacent and vice versa. The operation of summing over unit j is limited to the neighbors of unit i.

**Table S1. Major outbreaks of notifiable infectious diseases that occurred in China (mainland) between 2005 and 2020**

| Disease | Period | Region | Number of cases | Number of deaths |
| --- | --- | --- | --- | --- |
| H5N1 Avian Influenza | Oct. 2005 to Aug. 2006 | Multiple provinces, including Hunan, Guangxi, and Anhui provinces | Around 45 cases | 26 deaths |
| Hand, Foot, and Mouth Disease | Mar. 2008 to Jul. 2008 | Multiple provinces in China | Over 70,000 cases | 35 deaths |
| H1N1 Influenza (Swine Flu) | Apr. 2009 – Aug. 2010 | Nationwide outbreak | Approximately 120,940 | Over 800 deaths |
| H7N9 Avian Influenza | Mar. 2013 to Aug. 2014 | Eastern China, particularly in Zhejiang, Jiangsu, and Shanghai | 458 cases | 175 deaths |
| COVID-19 | Dec. 2019 to present | Global pandemic | 756 million (as of Feb, 2023) | 6.8 million (as of Feb, 2023) |

**Table S2. Classification of Notifiable Infectious Diseases in China**

| Class | Items |
| --- | --- |
| Class A | Plague, Cholera |
| Class B | Severe Acute Respiratory Syndrome (SARS), Acquired immunodeficiency syndrome (AIDS), Viral hepatitis (including A, B, C, D, E and unspecified), Poliomyelitis, Human infections of highly pathogenic avian influenza, human infections of H7N9 avian influenza, Measles, Epidemic hemorrhagic fever (EHF), Rabies, Epidemic Encephalitis B, Dengue fever, Anthrax, Bacterial and amoebic dysentery, Tuberculosis, Typhoid & paratyphoid (*typhoid and paratyphoid are counted as a single disease type in the law*) , Epidemic (meningococcal) meningitis, Pertussis, Diphtheria, Neonatal tetanus, Scarlet fever, Brucellosis, Gonorrhea, Syphilis, Leptospirosis, Schistosomiasis, Malaria, Corona Virus Disease 2019 (COVID-19) |
| Class C | Influenza, Mumps, Rubella, Acute hemorrhagic conjunctivitis (AHC), Leprosy, Typhus, Leishmaniasis, Echinococcosis, Filariasis, Other infectious diarrheal diseases, Hand, foot and mouth disease (HFMD) |

**Table S3. Classification of eight types of notifiable infectious diseases**

| Type | Diseases |
| --- | --- |
| Quarantinable diseases | Plague, Cholera, Hemorrhagic fever |
| Vaccine-preventable diseases | Measles, Pertussis, Neonatal tetanus |
| Gastrointestinal diseases | Bacterial and amoebic dysentery, Typhoid and Paratyphoid |
| Vector-borne diseases | Japanese encephalitis, Malaria, Dengue fever, schistosomiasis |
| Zoonotic infections | Leptospirosis, Brucellosis, Anthrax, Rabies |
| Bacterial infections | Epidemic cerebrospinal meningitis, Scarlet fever, tuberculosis |
| Sexually transmitted infections | Acquired immunodeficiency syndrome, Gonorrhea, Syphilis |
| Viral hepatitis | Hepatitis A, Hepatitis B, Hepatitis C, Hepatitis E, Unspecified hepatitis |

**Table S4.** Global Moran's I of 8 types of notifiable infectious diseases at the provincial level in 2005-2008, 2009-2012, 2013-2016 and 2017-2020.^a^

| Type | Infectious disease | 2005-2008 | | 2009-2012 | | 2013-2016 | | 2017-2020 | |
| --- | --- | --- | --- | --- | --- | --- | --- | --- | --- |
|  |  | **Moran's I** | ***P* value** | **Moran's I** | ***P* value** | **Moran's I** | ***P* value** | **Moran's I** | ***P* value** |
| Quarantinable diseases | | | | | | | | | |
|  | Plague | **0.054** | **.008** | **0.199** | **.003** | -0.087 | .09 | -0.003 | .27 |
|  | Cholera | **0.145** | **.02** | **0.197** | **.01** | 0.085 | .07 | **0.155** | **.001** |
|  | Hemorrhagic fever | **0.216** | **.004** | -0.081 | .30 | 0.053 | .20 | -0.056 | .40 |
| Vaccine-preventable diseases | | | | | | | | | |
|  | Measles | 0.124 | .06 | **0.313** | **.001** | **0.343** | **<.001** | **0.148** | **.001** |
|  | Pertussis | 0.059 | .09 | -0.012 | .37 | -0.030 | .47 | 0.071 | .16 |
|  | Neonatal tetanus | **0.201** | **.01** | **0.173** | **.03** | 0.066 | .12 | **0.234** | **.005** |
| Gastrointestinal diseases | | | | | | | | | |
|  | BAD | **0.226** | **.002** | **0.246** | **.001** | **0.253** | **.002** | **0.190** | **.006** |
|  | Typhoid and Paratyphoid | **0.209** | **.002** | **0.146** | **.004** | **0.142** | **.004** | **0.357** | **<.001** |
| Vector-borne diseases | | | | | | | | | |
|  | Japanese encephalitis | **0.462** | **<.001** | **0.460** | **<.001** | **0.364** | **<.001** | **0.246** | **.001** |
|  | Malaria | 0.001 | .35 | 0.064 | .15 | 0.113 | .06 | **0.164** | **.03** |
|  | Dengue fever | **0.076** | **.01** | 0.075 | .10 | 0.015 | .12 | 0.090 | .06 |
|  | Schistosomiasis | 0.008 | .13 | -0.031 | .47 | **0.201** | **.001** | 0.003 | .18 |
| Zoonotic infections | | | | | | | | | |
|  | Leptospirosis | **0.326** | **<.001** | **0.340** | **<.001** | **0.138** | **.006** | **0.386** | **<.001** |
|  | Brucellosis | **0.185** | **<.001** | **0.193** | **<.001** | **0.307** | **<.001** | **0.313** | **<.001** |
|  | Anthrax | **0.548** | **<.001** | **0.561** | **<.001** | **0.327** | **<.001** | **0.233** | **<.001** |
|  | Rabies | **0.397** | **<.001** | **0.331** | **<.001** | **0.286** | **.001** | **0.523** | **<.001** |
| Bacterial infections | | | | | | | | | |
|  | ECM | 0.092 | .10 | -0.004 | .34 | 0.017 | .06 | 0.025 | .17 |
|  | Scarlet fever | **0.386** | **<.001** | **0.415** | **<.001** | **0.382** | **<.001** | **0.306** | **.001** |
|  | Tuberculosis | **0.184** | **.02** | **0.311** | **<.001** | **0.362** | **<.001** | **0.383** | **<.001** |
| Sexually transmitted infections | | | | | | | | | |
|  | AIDS | 0.046 | .19 | **0.128** | **.03** | **0.276** | **.001** | **0.406** | **<.001** |
|  | Gonorrhea | **0.317** | **<.001** | **0.346** | **<.001** | **0.335** | **<.001** | **0.334** | **<.001** |
|  | Syphilis | **0.306** | **<.001** | **0.178** | **.02** | 0.068 | .16 | **0.235** | **.006** |
| Viral hepatitis | | | | | | | | | |
|  | Hepatitis A | **0.459** | **<.001** | **0.575** | **<.001** | **0.317** | **<.001** | **0.305** | **<.001** |
|  | Hepatitis B | **0.270** | **.002** | **0.221** | **.006** | 0.124 | .07 | **0.185** | **.02** |
|  | Hepatitis C | **0.250** | **.003** | **0.199** | **.01** | **0.180** | **.02** | **0.204** | **.01** |
|  | Hepatitis E | **0.324** | **<.001** | **0.406** | **<.001** | **0.309** | **.001** | **0.190** | **.02** |
|  | Unspecified hepatitis | **0.289** | **<.001** | **0.179** | **.007** | **0.161** | **.02** | 0.091 | .12 |

**Note:**

^a^ Numbers highlighted in bold denote *P* value < .05.

^b^ BAD=bacillary and amoebic dysentery; ECM=epidemic cerebrospinal meningitis; AIDS=acquired immune deficiency syndrome

**Figure S1.** Local Moran's I of 27 notifiable infectious diseases at the provincial level in 2005-2008.


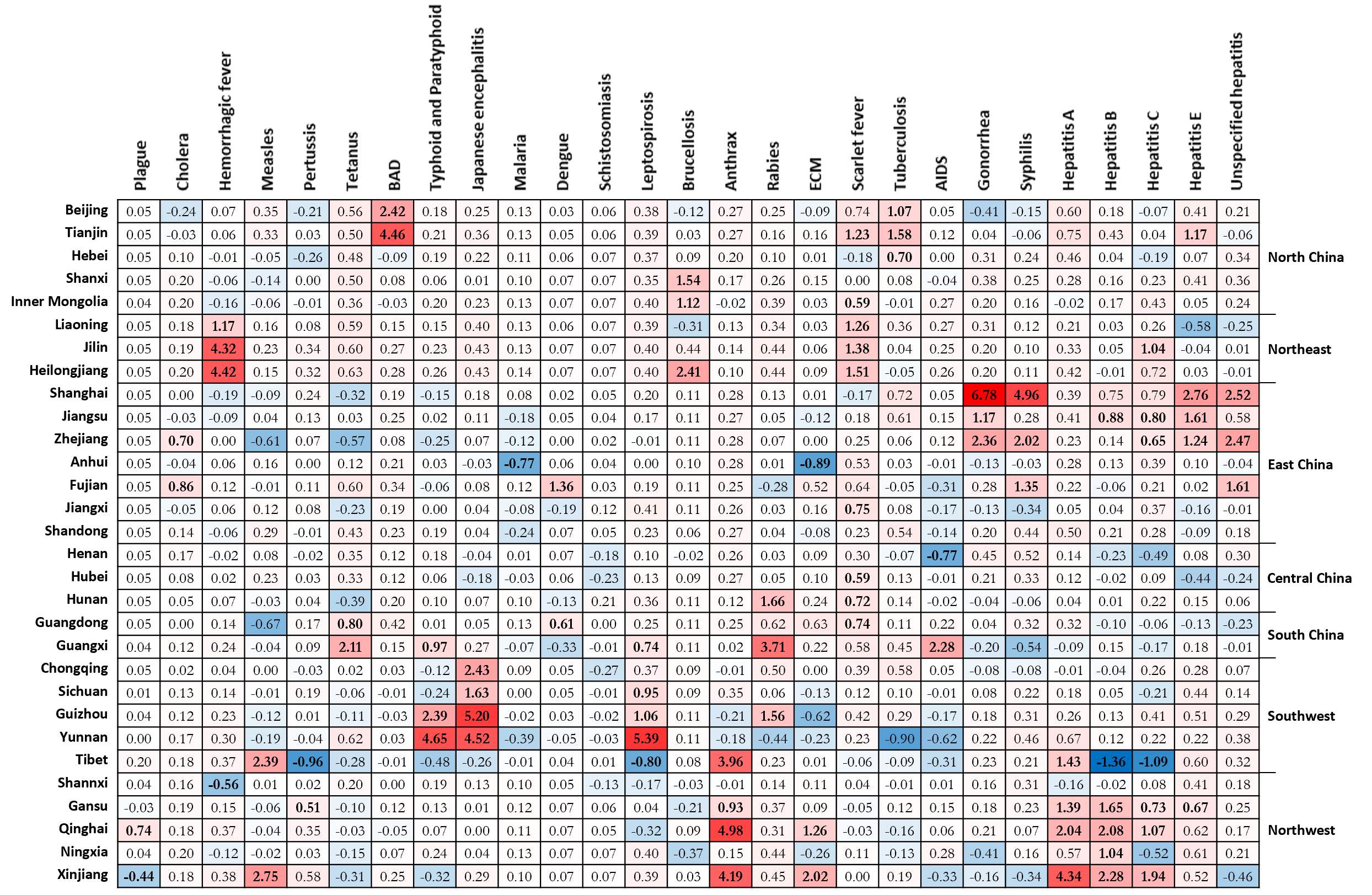


**Note:**

^a^ Numbers highlighted in bold denote *P* value < .05.

^b^ BAD=bacillary and amoebic dysentery; ECM=epidemic cerebrospinal meningitis; AIDS=acquired immune deficiency syndrome.

**Figure S2.** Local Moran's I of 27 notifiable infectious diseases at the provincial level in 2009-2012.


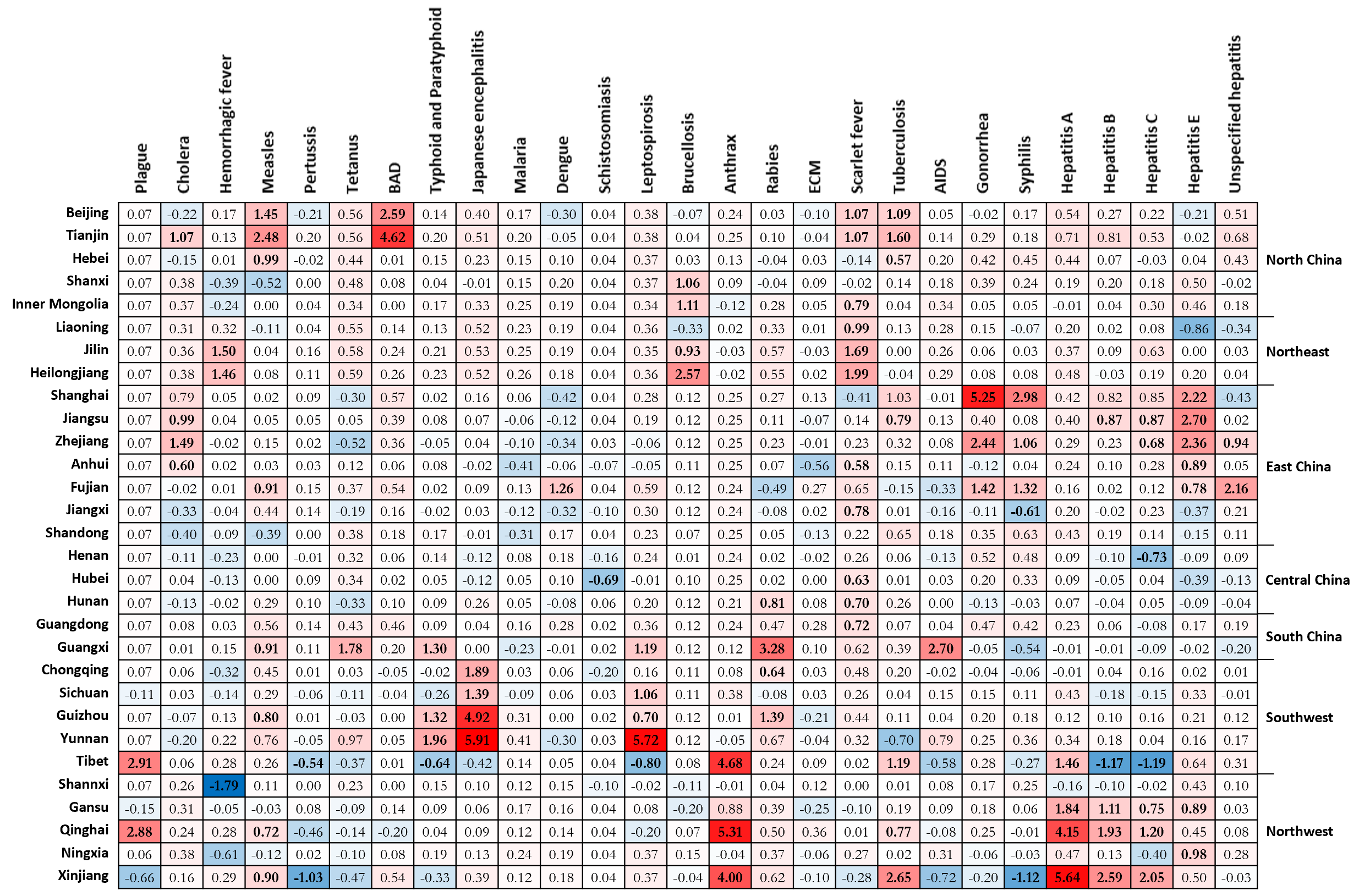


^a^ Numbers highlighted in bold denote *P* value < .05.

^b^ BAD=bacillary and amoebic dysentery; ECM=epidemic cerebrospinal meningitis; AIDS=acquired immune deficiency syndrome.

**Figure S3.** Local Moran's I of 26 notifiable infectious diseases at the provincial level in 2013-2016.


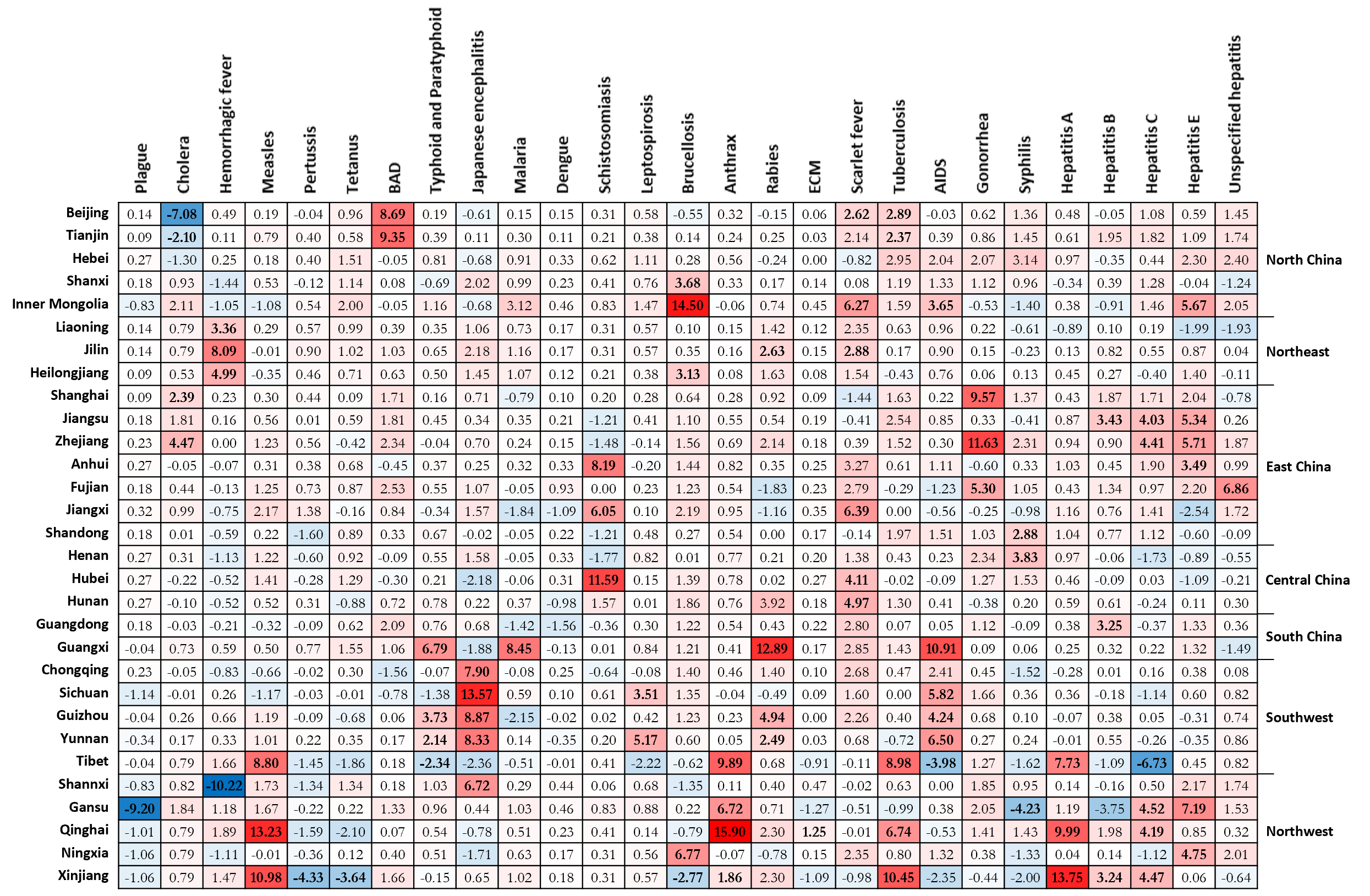


^a^ Numbers highlighted in bold denote *P* value < .05.

^b^ BAD=bacillary and amoebic dysentery; ECM=epidemic cerebrospinal meningitis; AIDS=acquired immune deficiency syndrome.

**Figure S4.** Descriptive maps and spatial cluster maps of Plague during 2005-2008, 2009-2012, 2013-2016, 2017-2020.


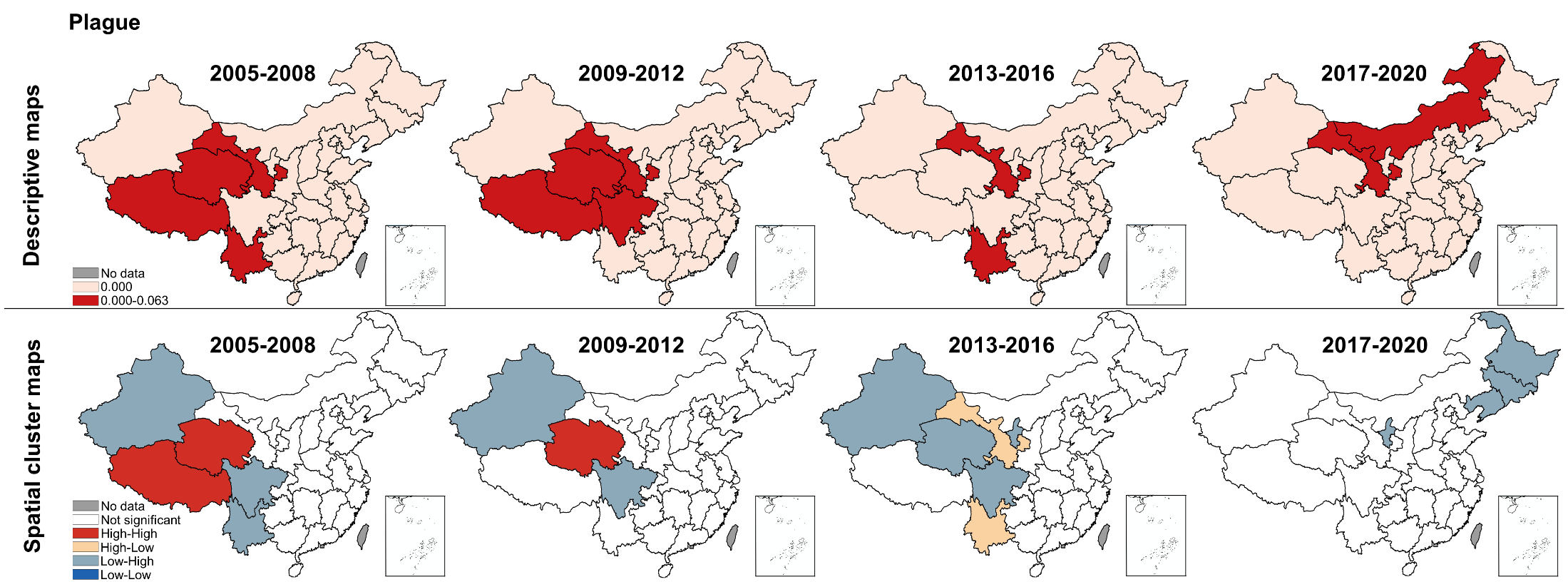


**Figure S5.** Descriptive maps and spatial cluster maps of Cholera during 2005-2008, 2009-2012, 2013-2016, 2017-2020.


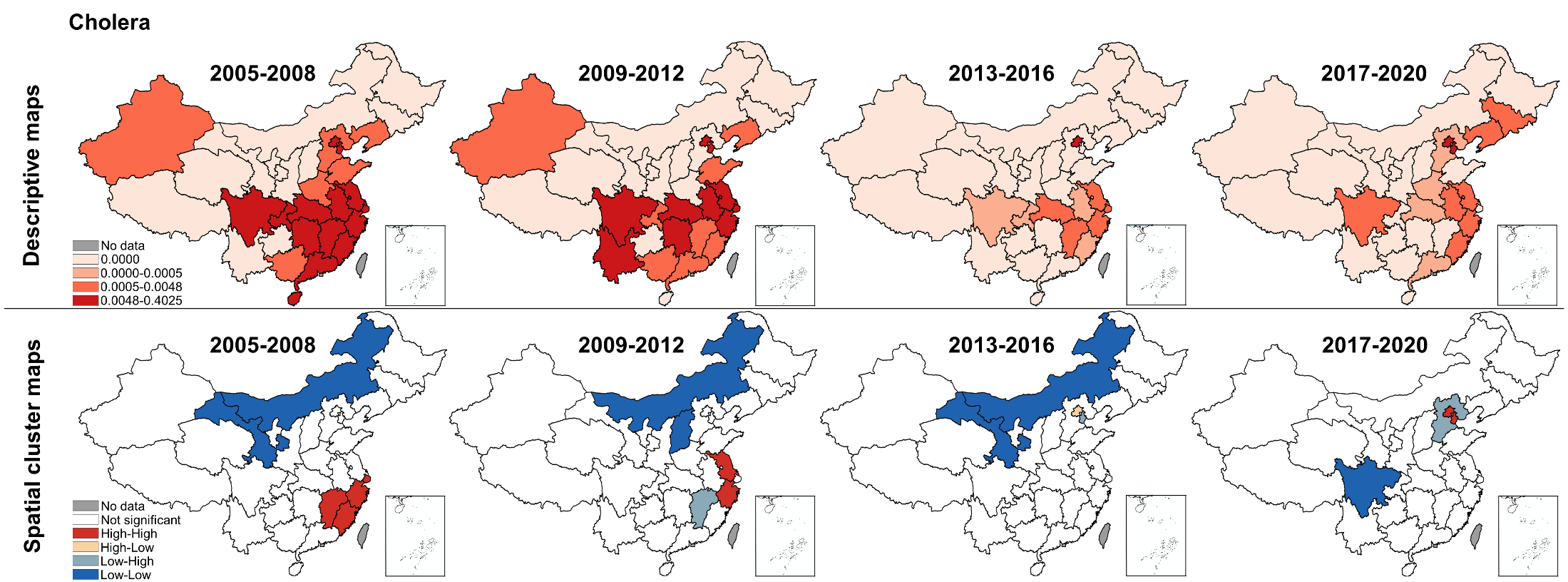


**Figure S6.** Descriptive maps and spatial cluster maps of Hemorrhagic fever during 2005-2008, 2009-2012, 2013-2016, 2017-2020.


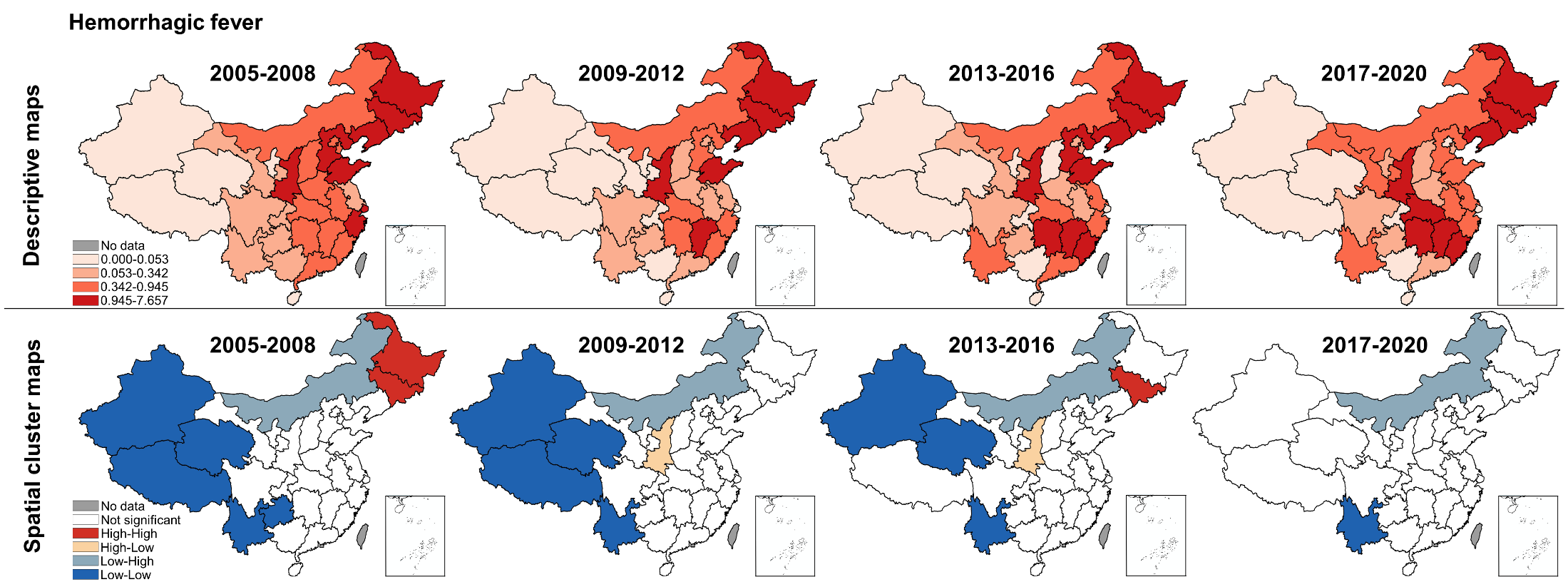


**Figure S7.** Descriptive maps and spatial cluster maps of Measles during 2005-2008, 2009-2012, 2013-2016, 2017-2020.


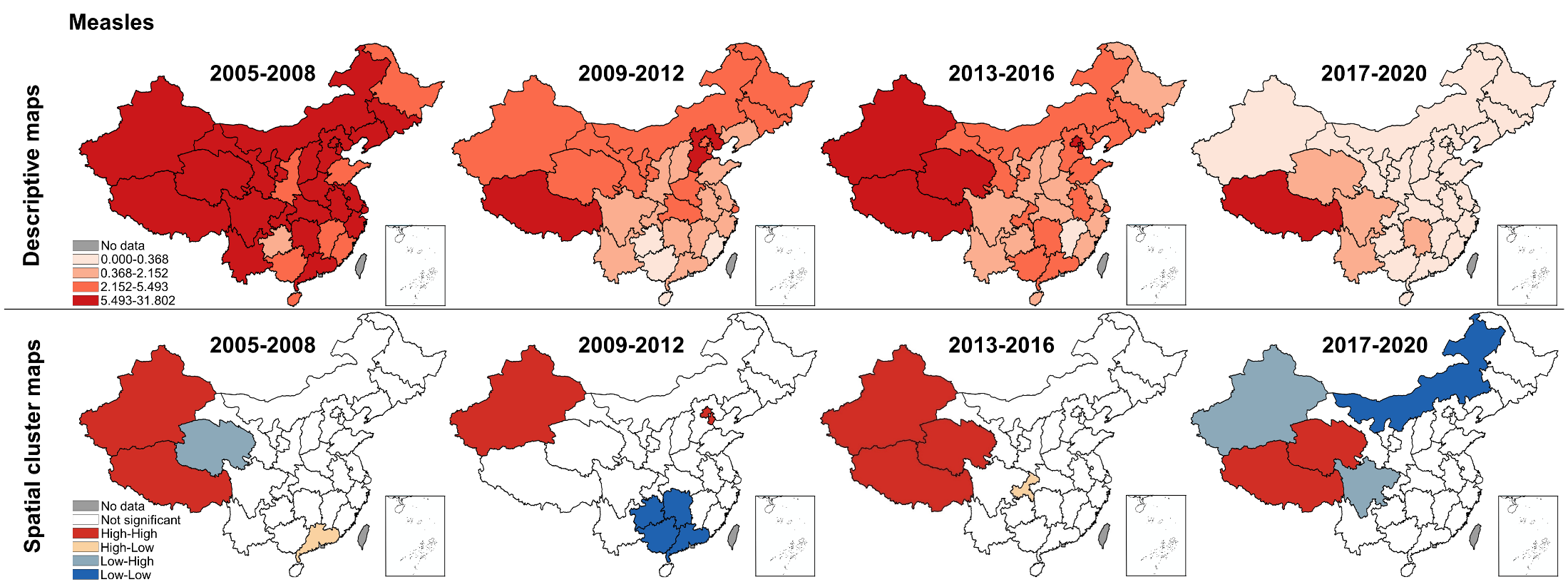


**Figure S8.** Descriptive maps and spatial cluster maps of Pertussis during 2005-2008, 2009-2012, 2013-2016, 2017-2020.


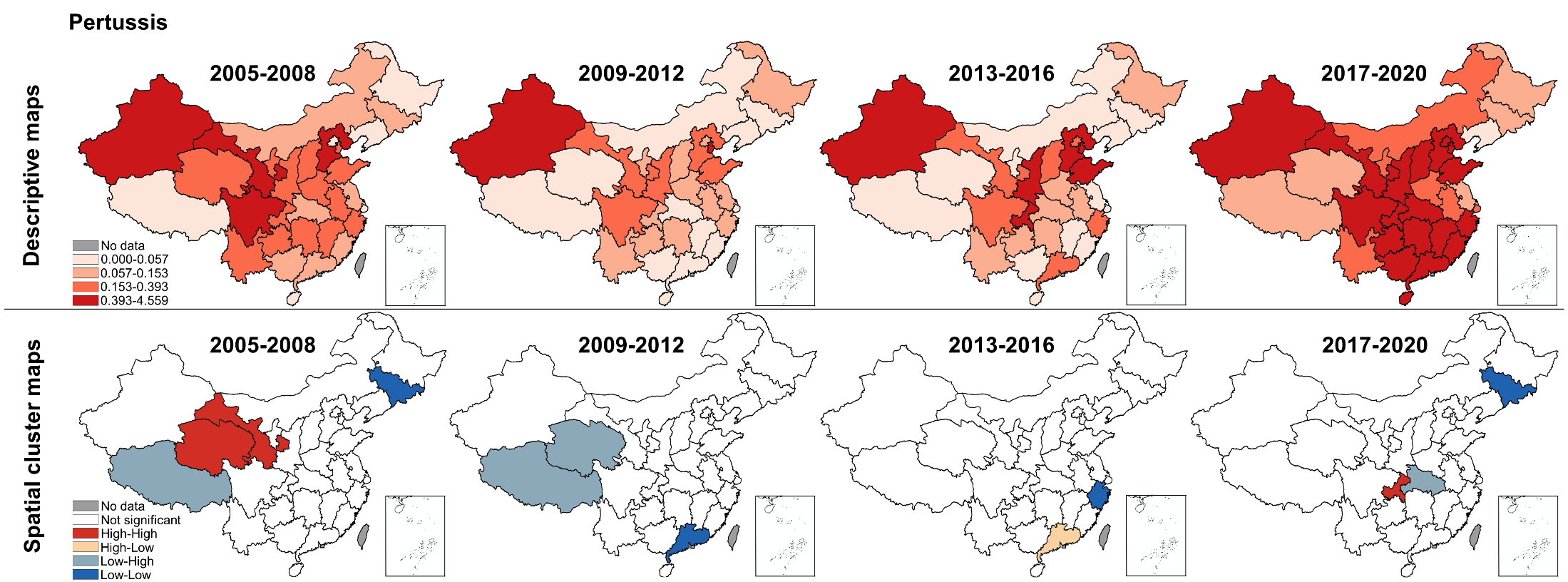


**Figure S9.** Descriptive maps and spatial cluster maps of Neonatal tetanus during 2005-2008, 2009-2012, 2013-2016, 2017-2020.


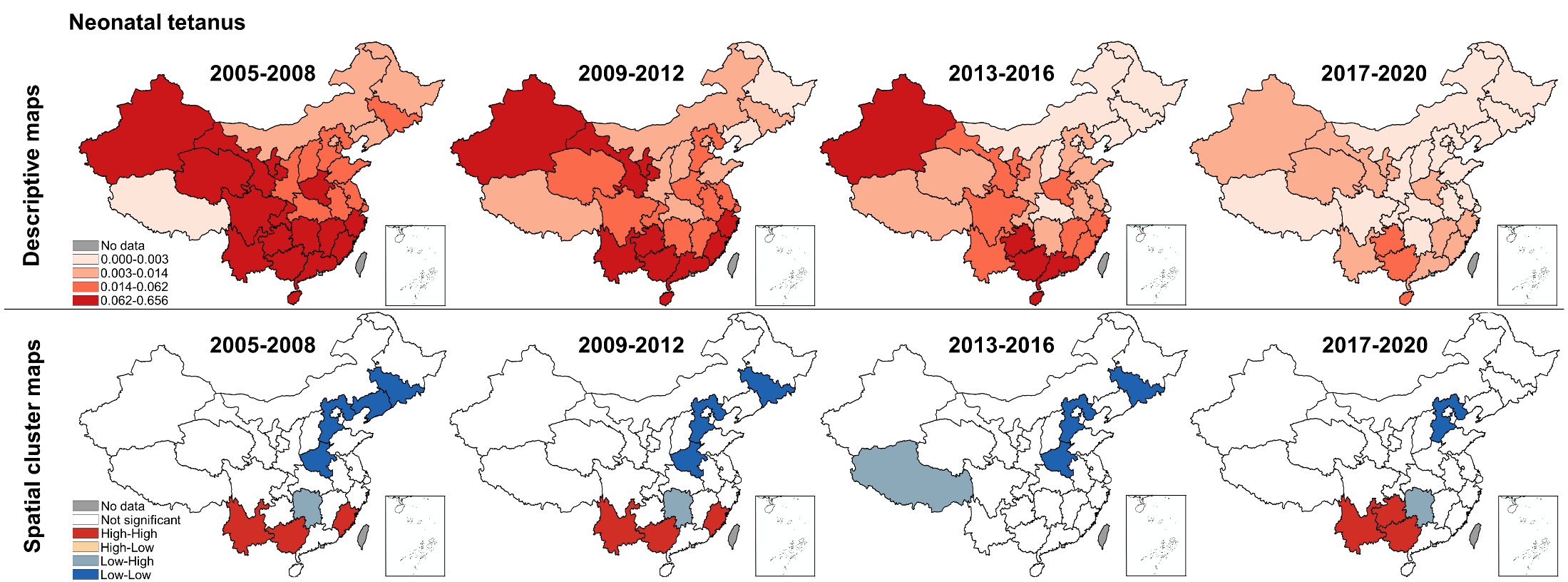


**Figure S10.** Descriptive maps and spatial cluster maps of Bacillary and amoebic dysentery (BAD) during 2005-2008, 2009-2012, 2013-2016, 2017-2020.


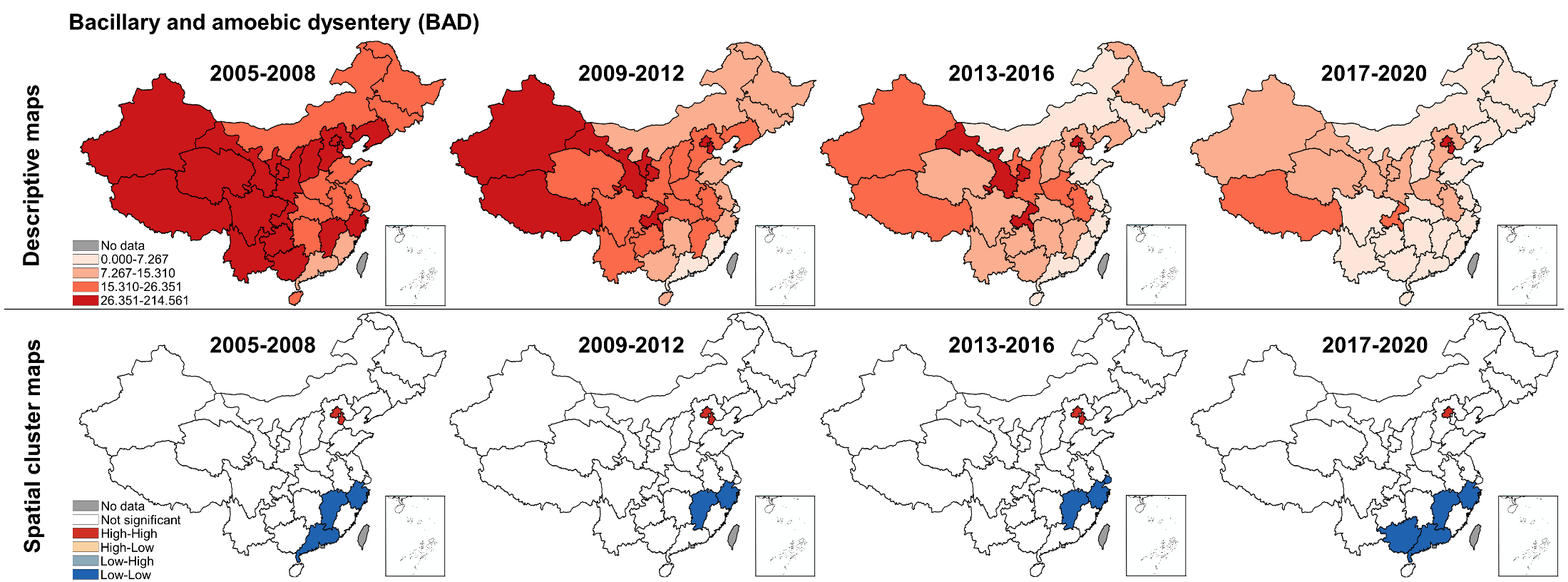


**Figure S11.** Descriptive maps and spatial cluster maps of Typhoid and Paratyphoid during 2005-2008, 2009-2012, 2013-2016, 2017-2020.


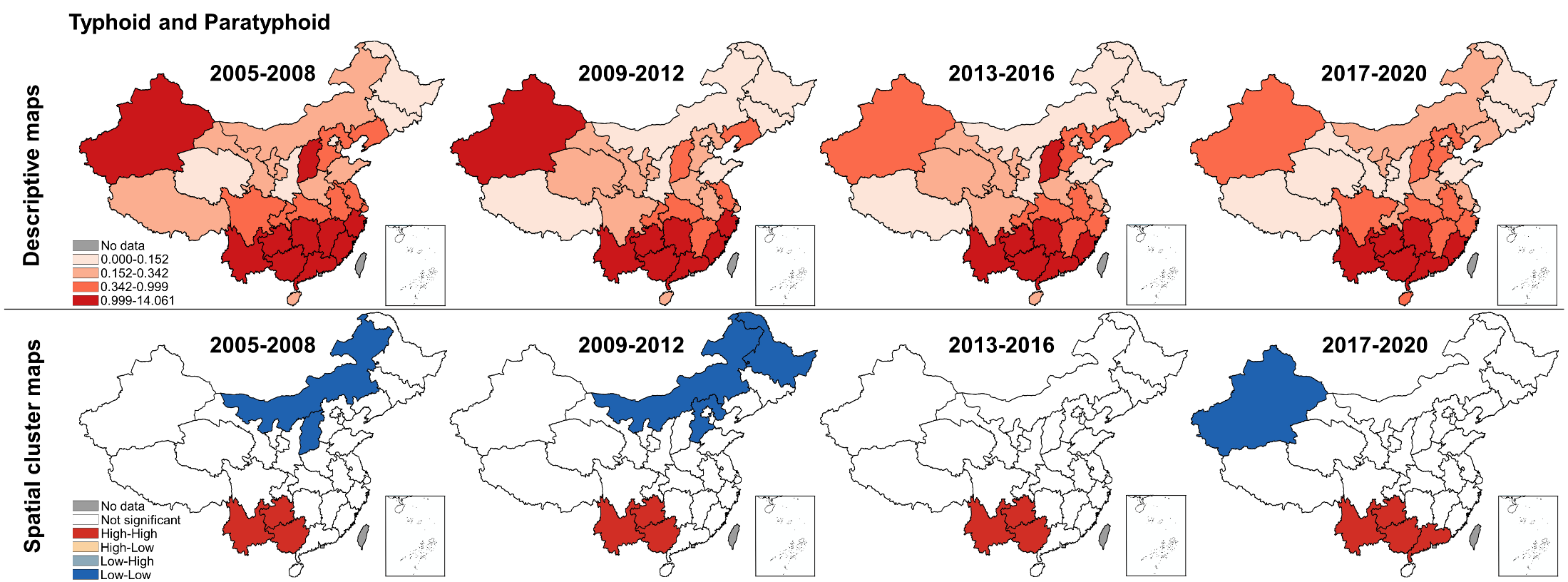


**Figure S12.** Descriptive maps and spatial cluster maps of Japanese encephalitis during 2005-2008, 2009-2012, 2013-2016, 2017-2020.


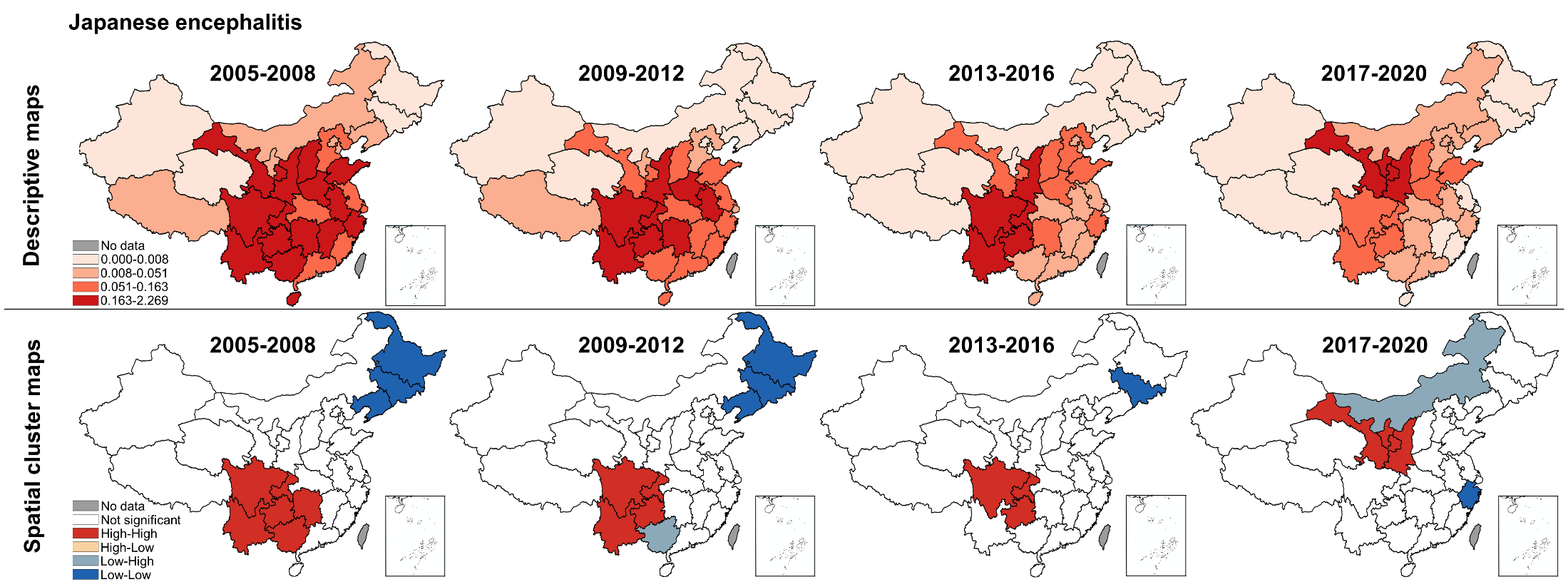


**Figure S13.** Descriptive maps and spatial cluster maps of Malaria during 2005-2008, 2009-2012, 2013-2016, 2017-2020.


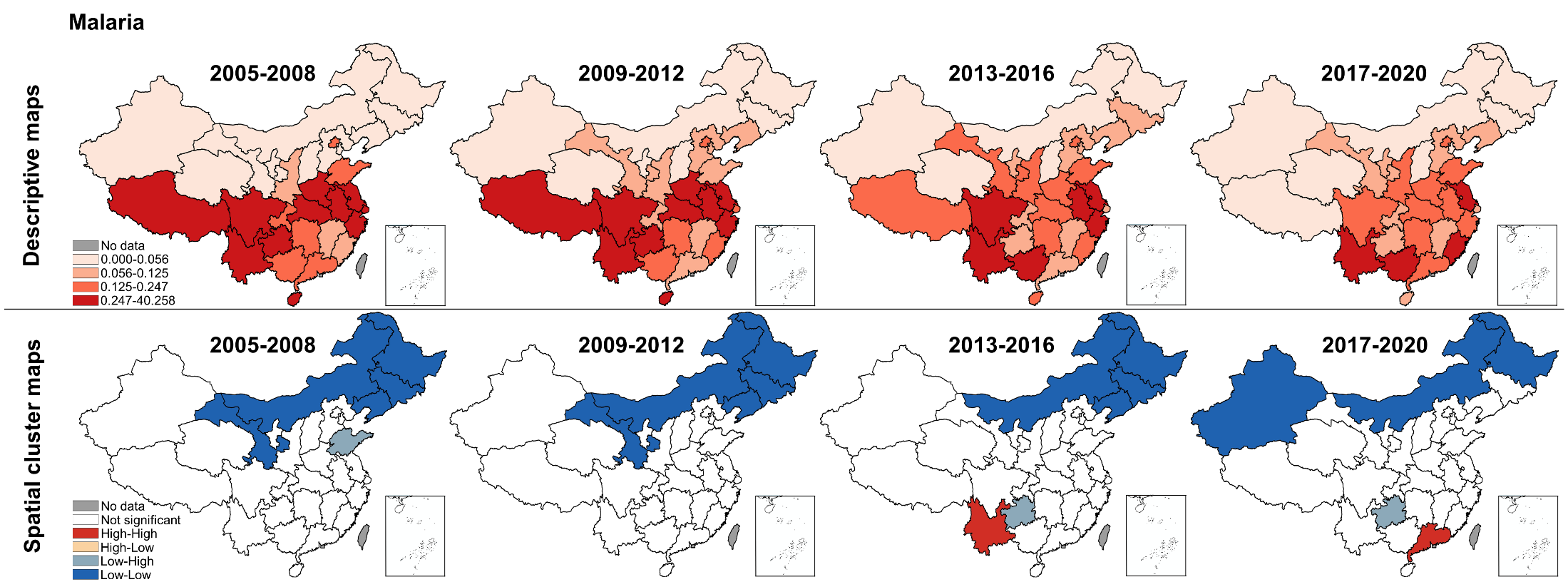


**Figure S14.** Descriptive maps and spatial cluster maps of Dengue fever during 2005-2008, 2009-2012, 2013-2016, 2017-2020.


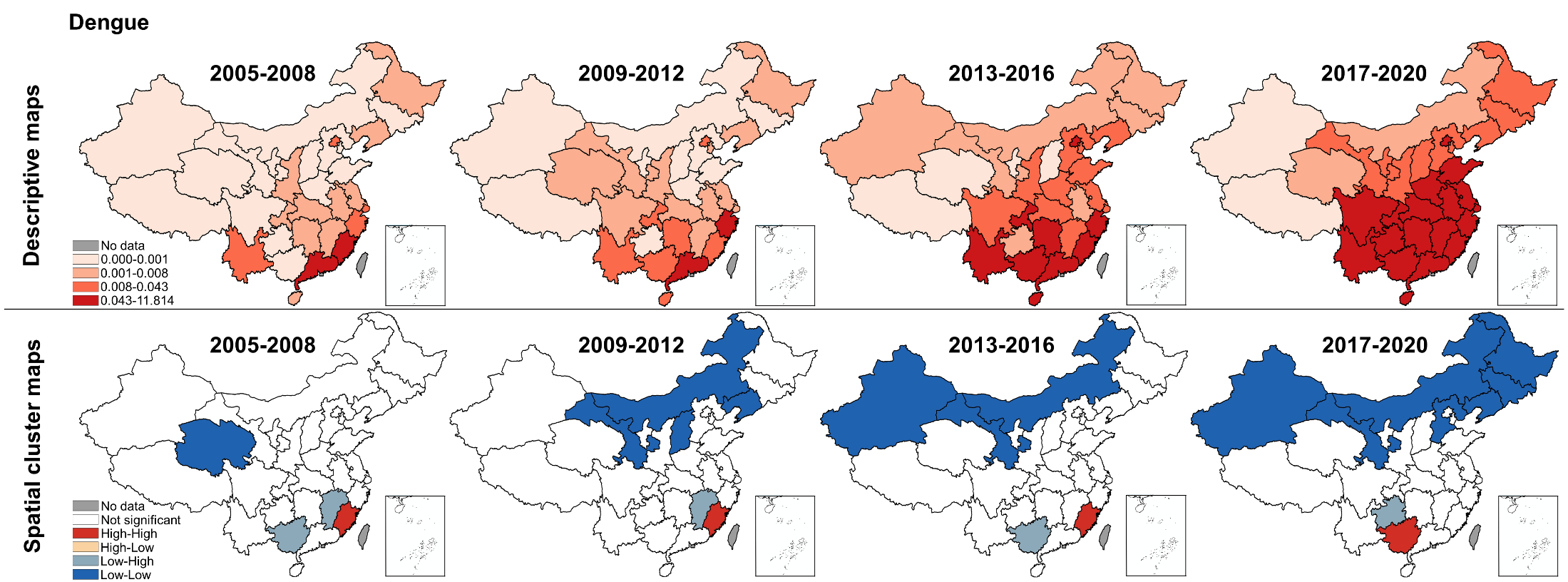


**Figure S15.** Descriptive maps and spatial cluster maps of Schistosomiasis during 2005-2008, 2009-2012, 2013-2016, 2017-2020.


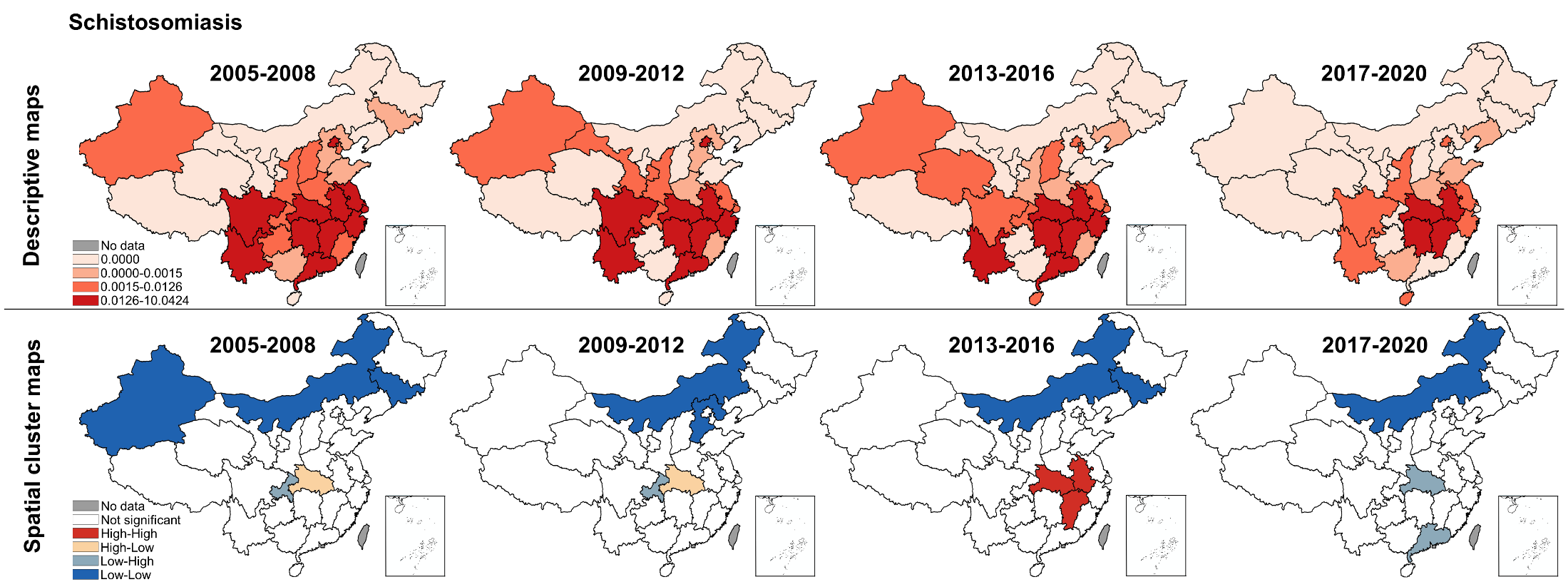


**Figure S16.** Descriptive maps and spatial cluster maps of Leptospirosis during 2005-2008, 2009-2012, 2013-2016, 2017-2020.


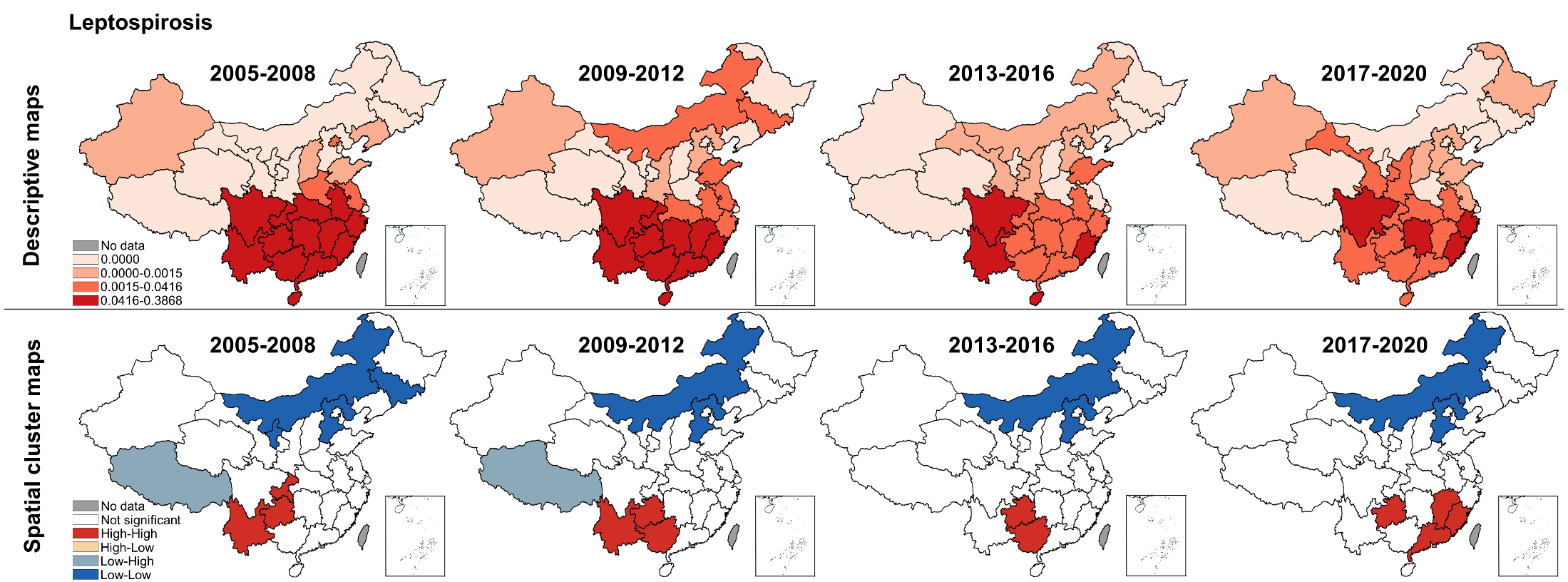


**Figure S17.** Descriptive maps and spatial cluster maps of Brucellosis during 2005-2008, 2009-2012, 2013-2016, 2017-2020.


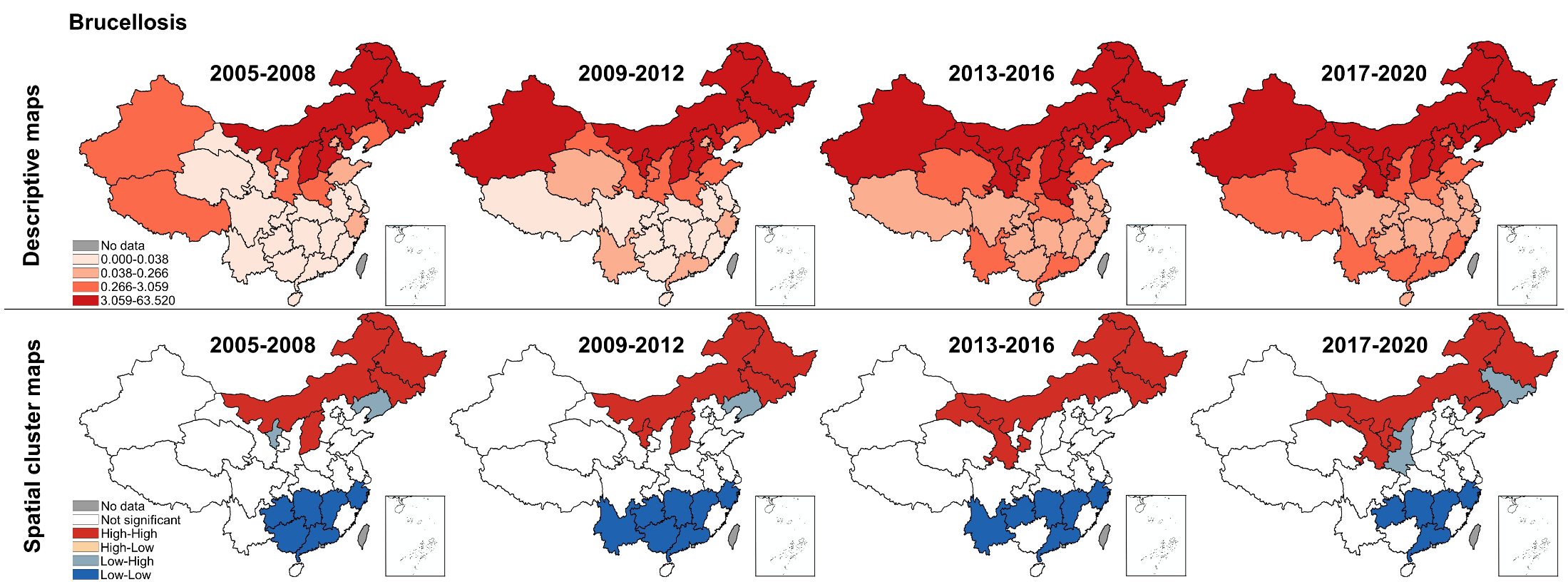


**Figure S18.** Descriptive maps and spatial cluster maps of Anthrax during 2005-2008, 2009-2012, 2013-2016, 2017-2020.


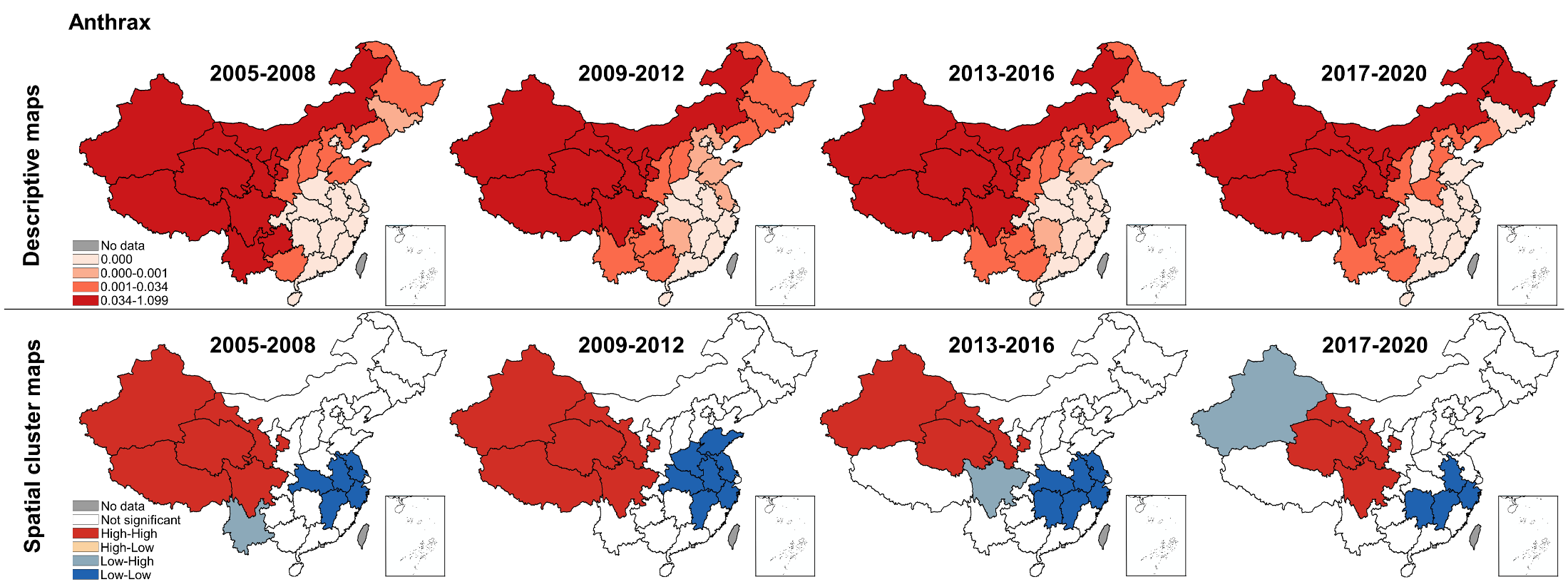


**Figure S19.** Descriptive maps and spatial cluster maps of Rabies during 2005-2008, 2009-2012, 2013-2016, 2017-2020.


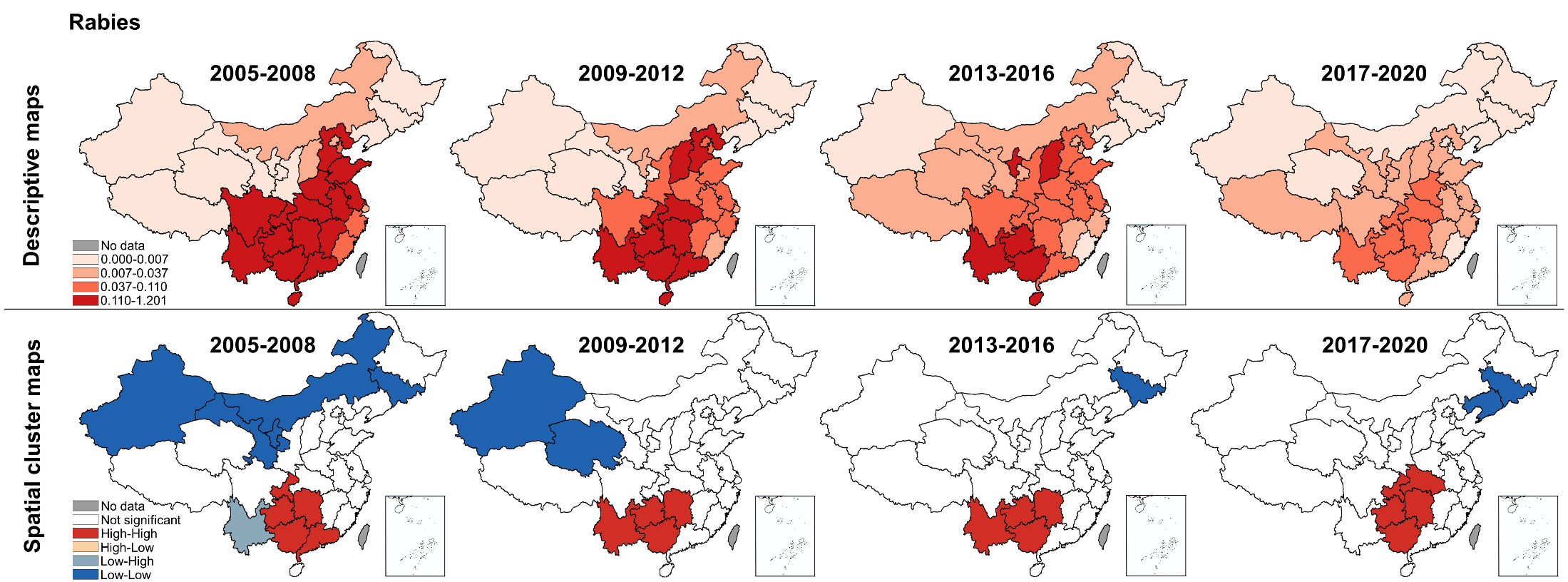


**Figure S20.** Descriptive maps and spatial cluster maps of Epidemic cerebrospinal meningitis (ECM) during 2005-2008, 2009-2012, 2013-2016, 2017-2020.


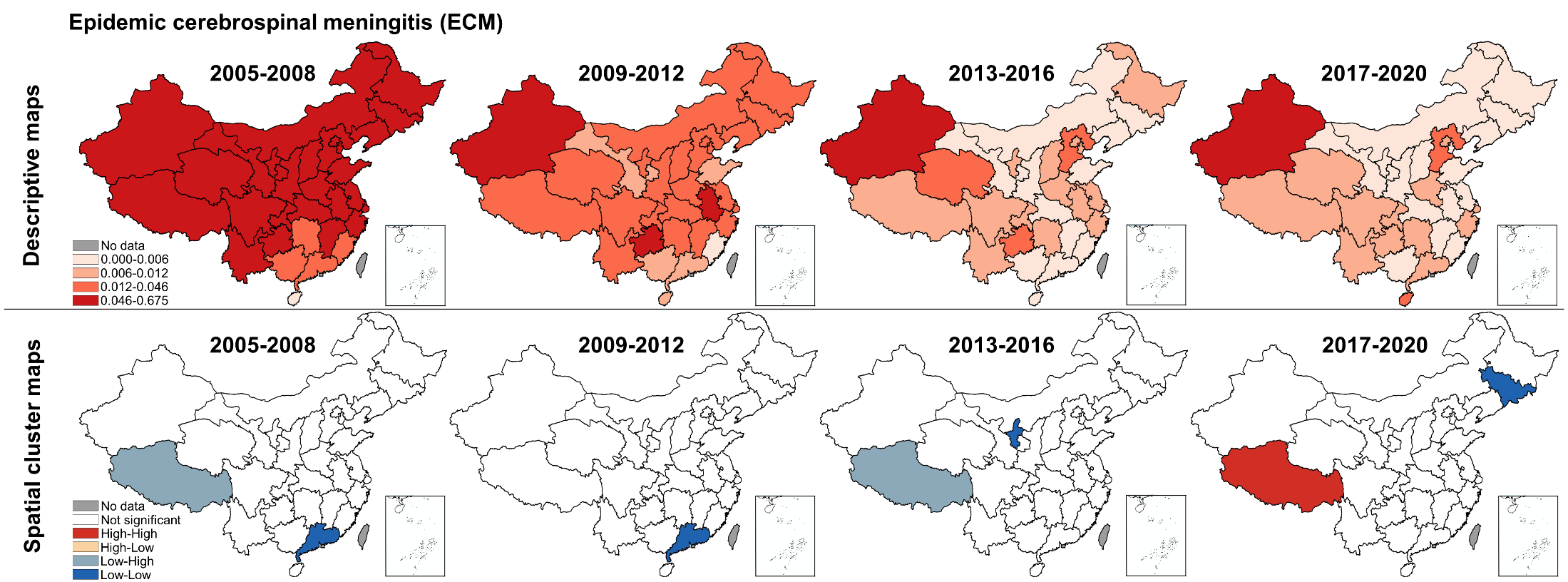


**Figure S21.** Descriptive maps and spatial cluster maps of Scarlet fever during 2005-2008, 2009-2012, 2013-2016, 2017-2020.


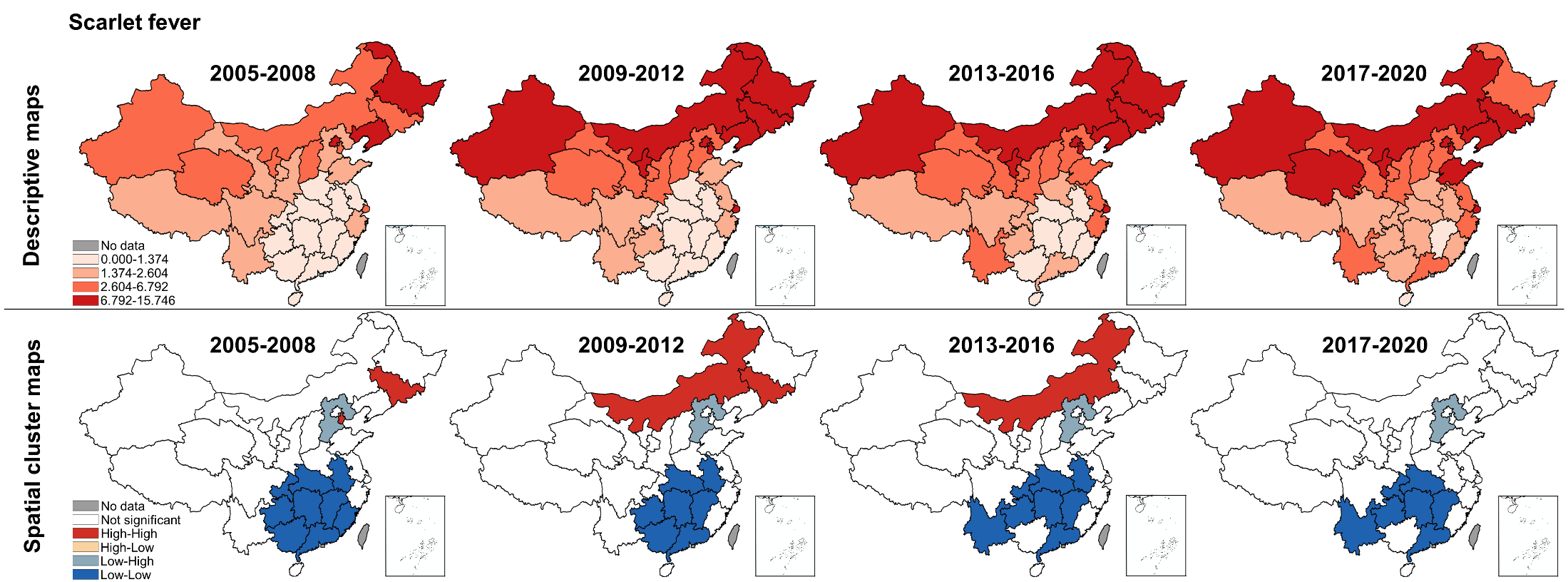


**Figure S22.** Descriptive maps and spatial cluster maps of Tuberculosis during 2005-2008, 2009-2012, 2013-2016, 2017-2020.


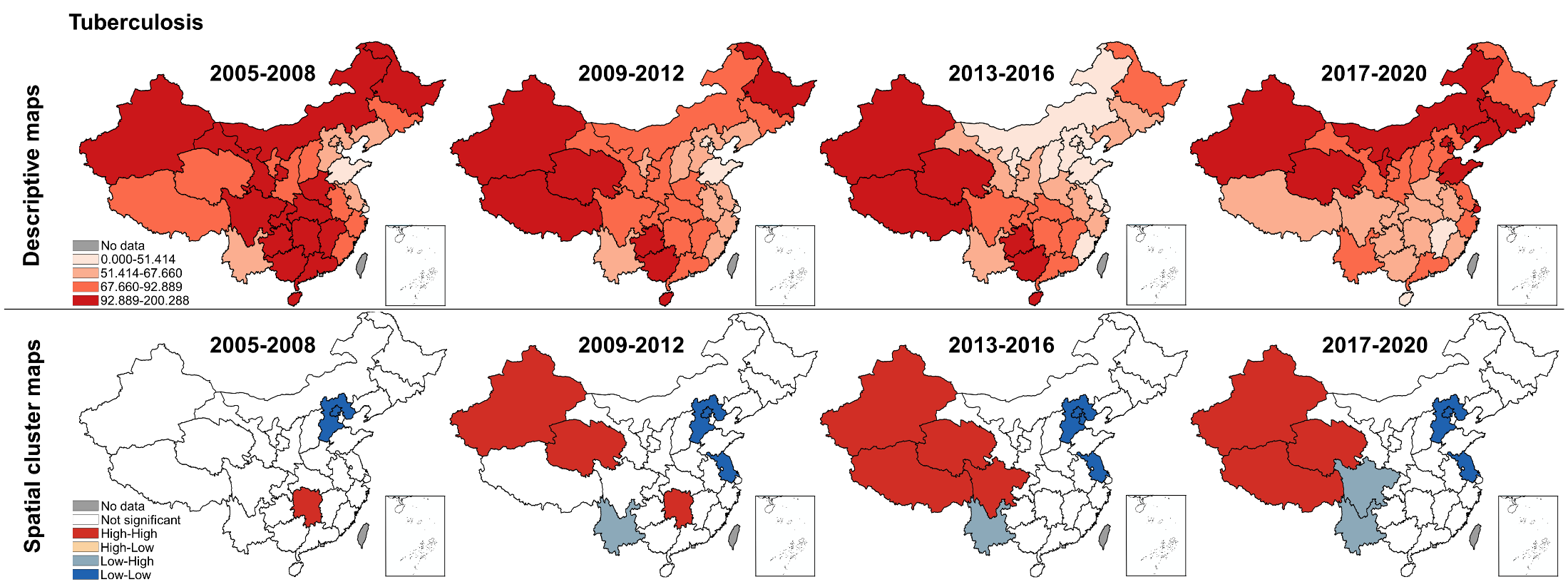


**Figure S23.** Descriptive maps and spatial cluster maps of Acquired immunodeficiency syndrome (AIDS) during 2005-2008, 2009-2012, 2013-2016, 2017-2020.


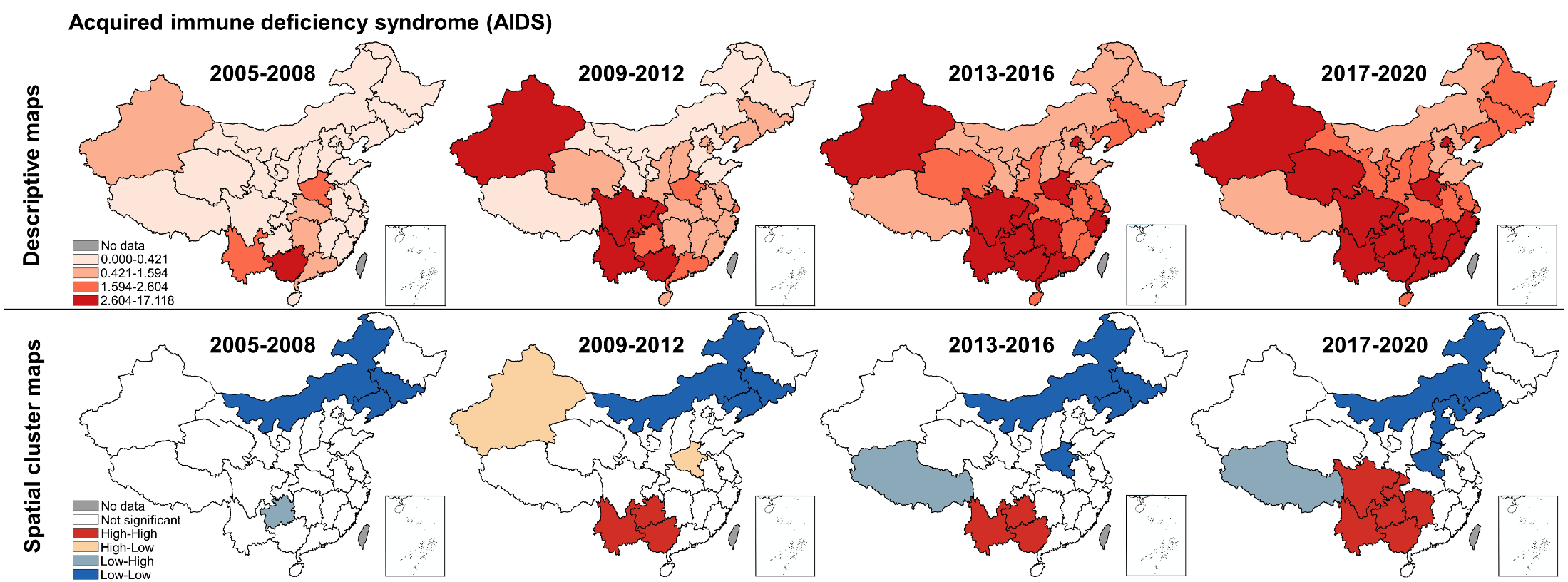


**Figure S24.** Descriptive maps and spatial cluster maps of Gonorrhea during 2005-2008, 2009-2012, 2013-2016, 2017-2020.


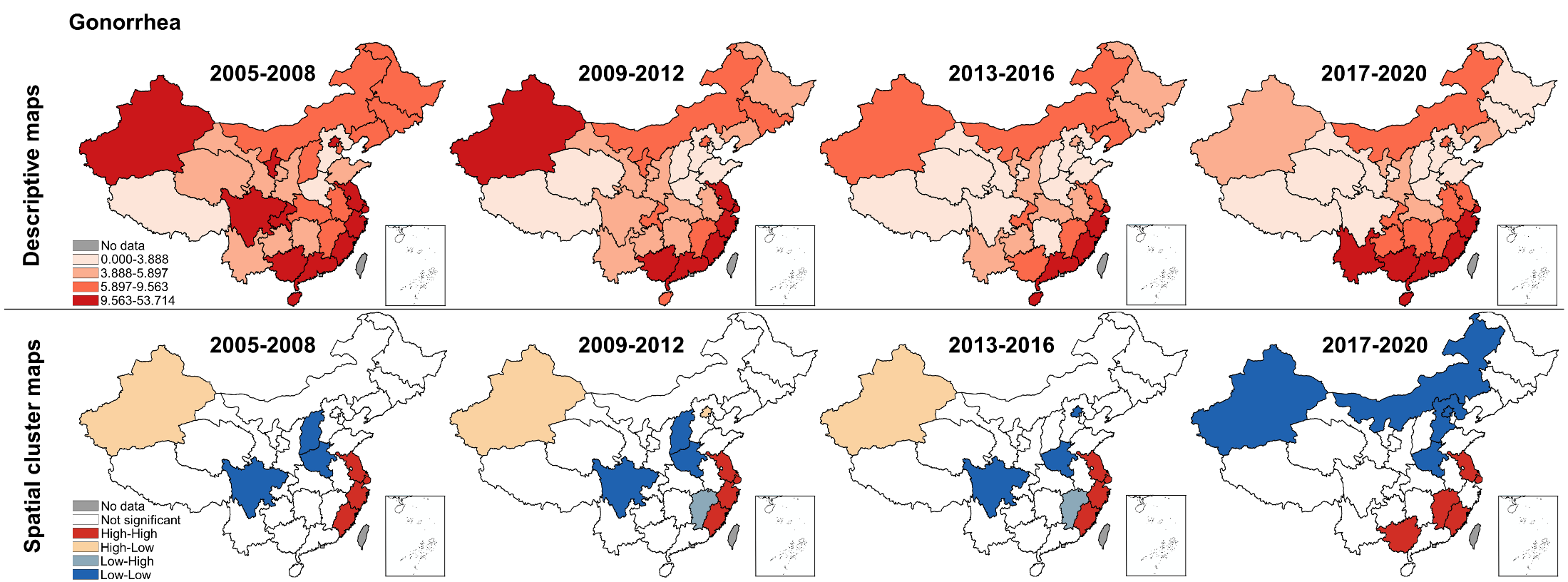


**Figure S25.** Descriptive maps and spatial cluster maps of Syphilis during 2005-2008, 2009-2012, 2013-2016, 2017-2020.


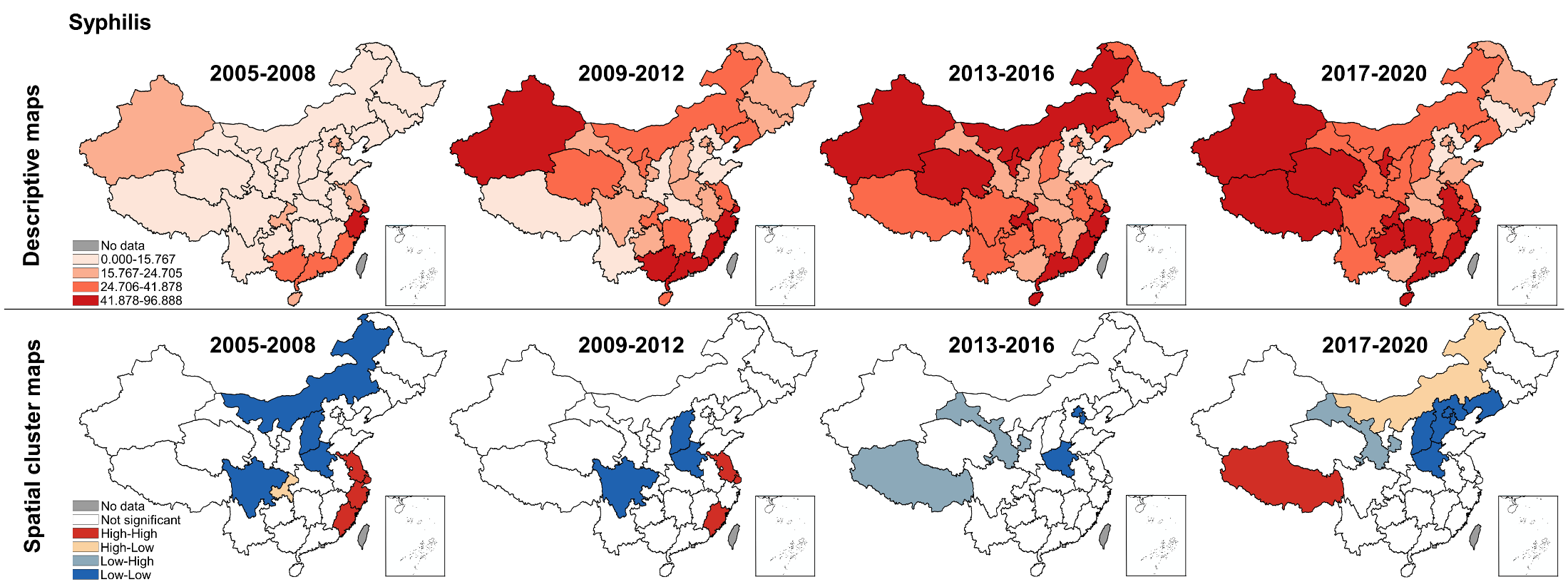


**Figure S26.** Descriptive maps and spatial cluster maps of Hepatitis A during 2005-2008, 2009-2012, 2013-2016, 2017-2020.


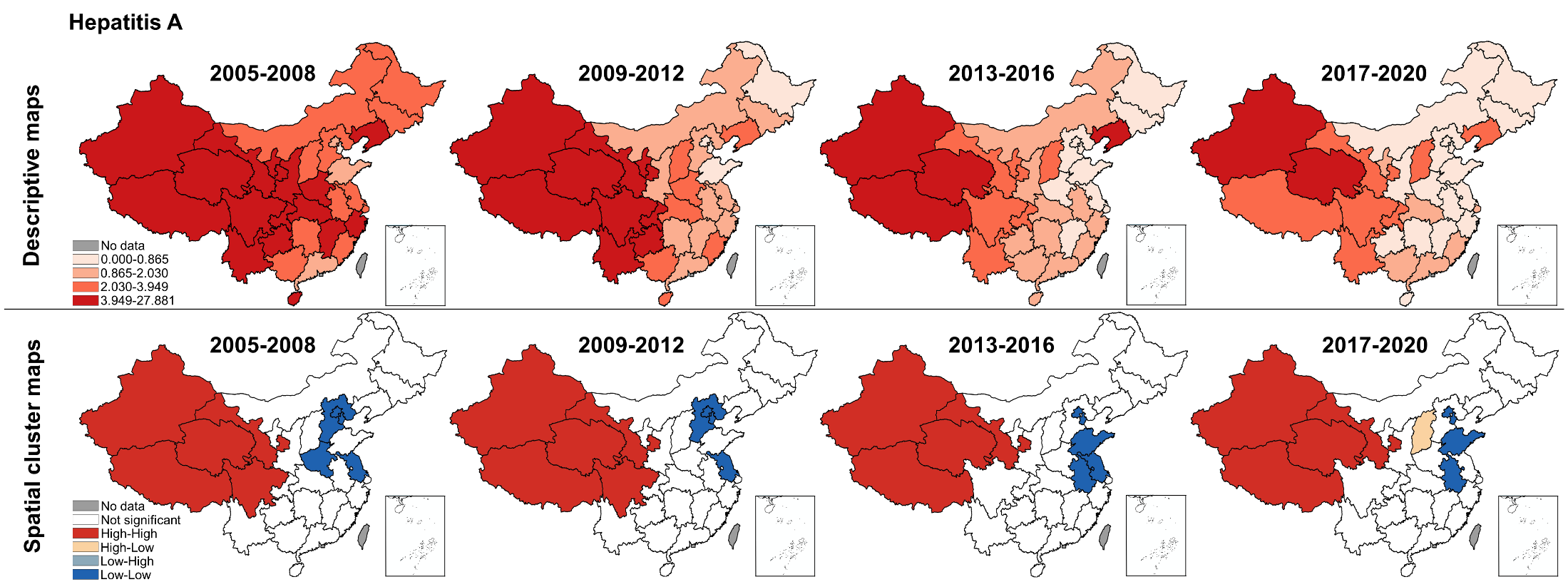


**Figure S27.** Descriptive maps and spatial cluster maps of Hepatitis B during 2005-2008, 2009-2012, 2013-2016, 2017-2020.


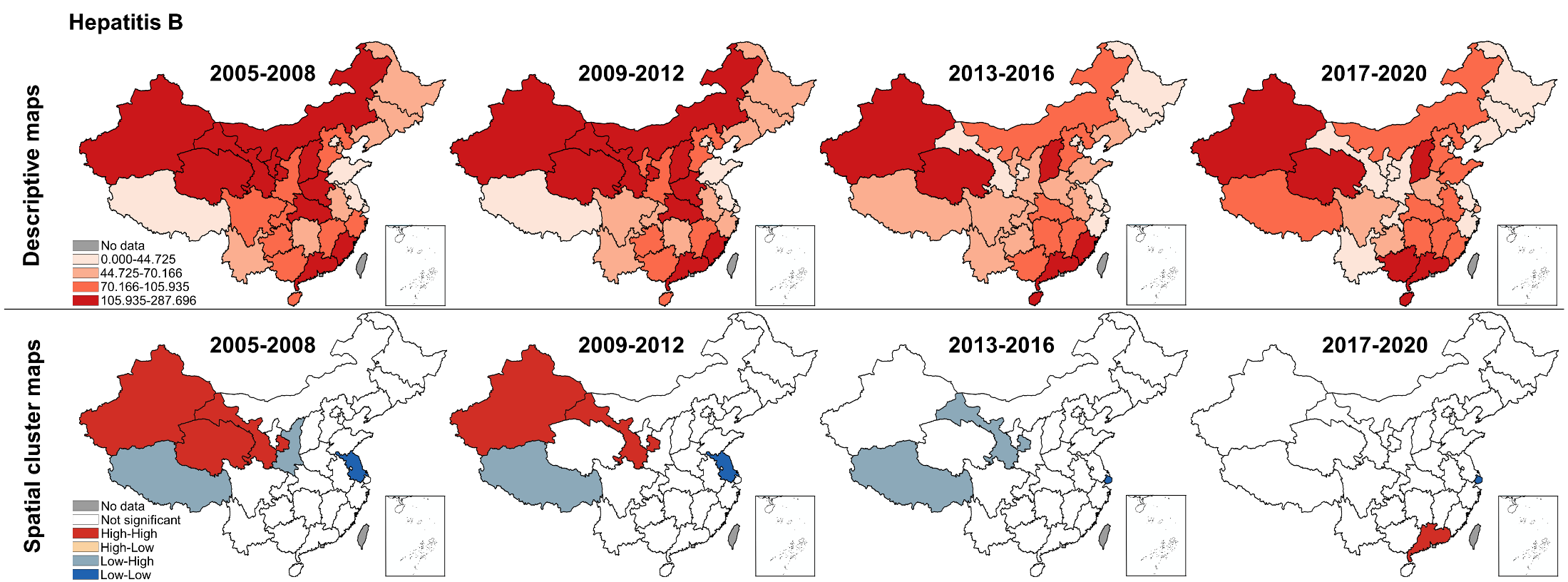


**Figure S28.** Descriptive maps and spatial cluster maps of Hepatitis C during 2005-2008, 2009-2012, 2013-2016, 2017-2020.


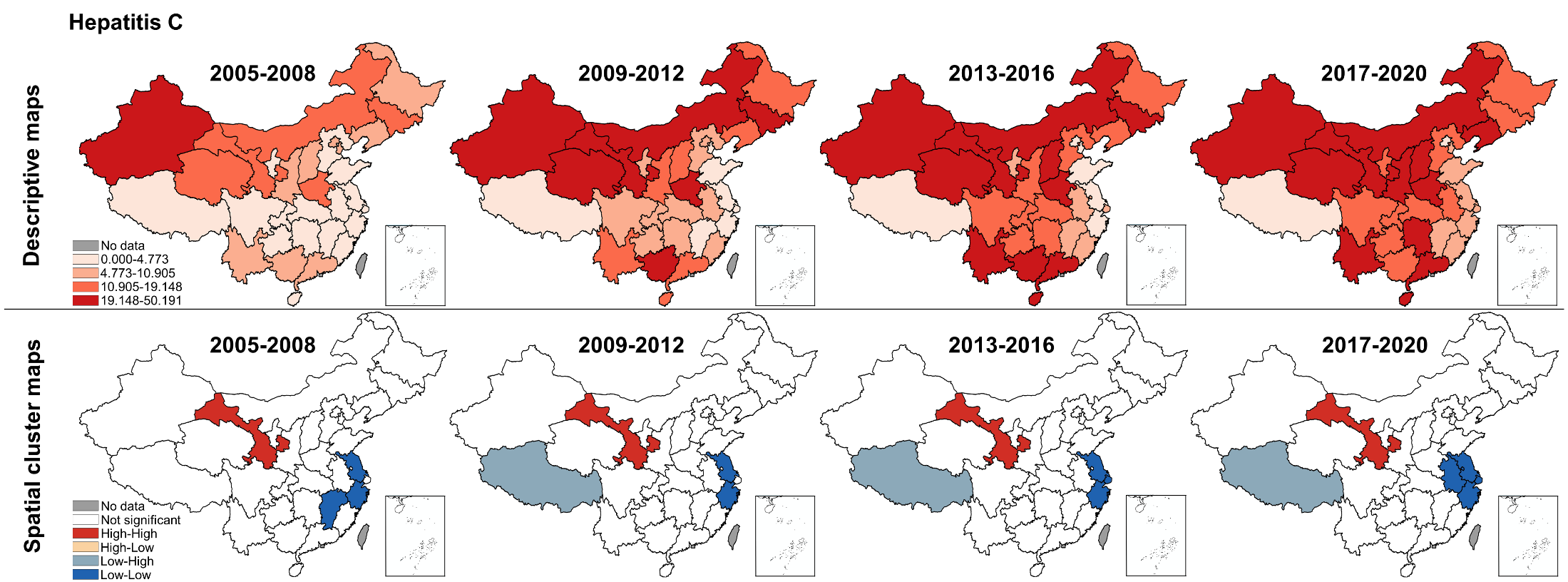


**Figure S29.** Descriptive maps and spatial cluster maps of Hepatitis E during 2005-2008, 2009-2012, 2013-2016, 2017-2020.


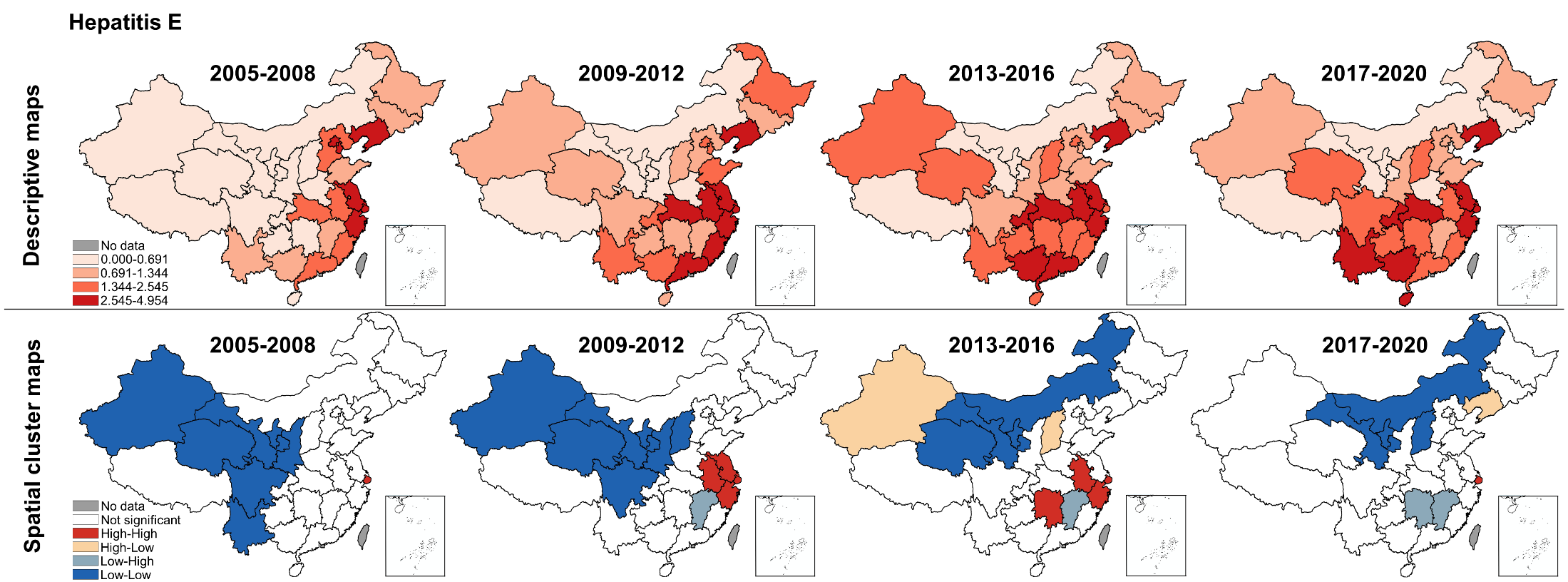


**Figure S30.** Descriptive maps and spatial cluster maps of Unspecified hepatitis during 2005-2008, 2009-2012, 2013-2016, 2017-2020.


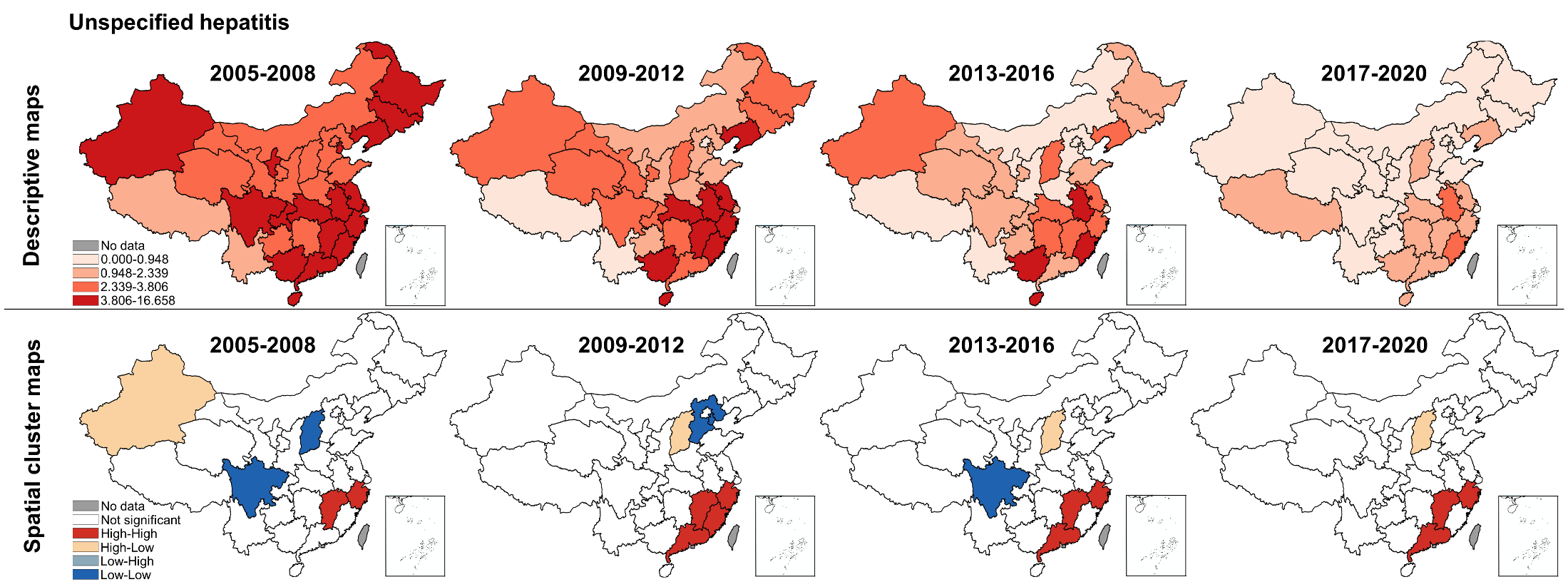

Supplement: Multimedia Appendix 1 [file publichealth_v9i1e42820_app1.docx]
